# Coarse-graining Hamiltonian systems using WSINDy

**DOI:** 10.1038/s41598-024-64730-0

**Published:** 2024-06-24

**Authors:** Daniel A. Messenger, Joshua W. Burby, David M. Bortz

**Affiliations:** 1 Department of Applied Mathematics, University of Colorado, Boulder, CO 80309-0526 USA; 2 Department of Physics and Institute for Fusion Studies, The University of Texas at Austin, Austin, TX 78712 USA

**Keywords:** WSINDy, Hamiltonian systems, Coarse-graining, Mathematics and computing, Applied mathematics, Computational science, Plasma physics

## Abstract

Weak form equation learning and surrogate modeling has proven to be computationally efficient and robust to measurement noise in a wide range of applications including ODE, PDE, and SDE discovery, as well as in coarse-graining applications, such as homogenization and mean-field descriptions of interacting particle systems. In this work we extend this coarse-graining capability to the setting of Hamiltonian dynamics which possess approximate symmetries associated with timescale separation. A smooth $\varepsilon$-dependent Hamiltonian vector field $X_\varepsilon$ possesses an approximate symmetry if the limiting vector field $X_0=\lim _{\varepsilon \rightarrow 0}X_\varepsilon$ possesses an exact symmetry. Such approximate symmetries often lead to the existence of a Hamiltonian system of reduced dimension that may be used to efficiently capture the dynamics of the symmetry-invariant dependent variables. Deriving such reduced systems, or approximating them numerically, is an ongoing challenge. We demonstrate that WSINDy can successfully identify this reduced Hamiltonian system in the presence of large perturbations imparted in the $\varepsilon >0$ regime, while remaining robust to extrinsic noise. This is significant in part due to the nontrivial means by which such systems are derived analytically. WSINDy naturally preserves the Hamiltonian structure by restricting to a trial basis of Hamiltonian vector fields. The methodology is computationally efficient, often requiring only a single trajectory to learn the global reduced Hamiltonian, and avoiding forward solves in the learning process. In this way, we argue that weak-form equation learning is particularly well-suited for Hamiltonian coarse-graining. Using nearly-periodic Hamiltonian systems as a prototypical class of systems with approximate symmetries, we show that WSINDy robustly identifies the correct leading-order system, with dimension reduced by at least two, upon observation of the relevant degrees of freedom. While our main contribution is computational, we also provide a contribution to the literature on averaging theory by proving that first-order averaging at the level of vector fields preserves Hamiltonian structure in nearly-periodic Hamiltonian systems. This provides theoretical justification for our approach as WSINDy’s computations occur at the level of Hamiltonian vector fields. We illustrate the efficacy of our proposed method using physically relevant examples, including coupled oscillator dynamics, the Hénon–Heiles system for stellar motion within a galaxy, and the dynamics of charged particles.

## Introduction

Hamiltonian mechanics, a formulation of classical mechanics, is used to describe non-dissipative systems^1^ in terms of a conserved quantity known as the *Hamiltonian*. Hamiltonian descriptions of physical systems reveal geometric properties not immediately present in Newtonian or Lagrangian formulations. Expression of the dynamics in terms of a conserved quantity is also essential in the formulation of quantum mechanics. In fact, the Schrödinger equation comprises an infinite-dimensional Hamiltonian system^2^. The geometry of phase space allows one to systematically explore conserved quantities, also known as constants of motion, which indicate the presence of *symmetry*. A symmetry is map on phase space which commutes with the flow-map of the dynamics, and may be used to reduce the size of phase space. Continuous families of symmetries lead to phase space dimension reduction. While many physical systems do possess quantities which are strictly conserved, often the system possesses *approximately conserved quantities* which lead to *approximate symmetries*. Such approximate symmetries can still be used to derive a reduced-order system that approximates well the important degrees of freedom in the original system, but such derivations remain an ongoing challenge.

An important example of a system with an approximate symmetry is a charged particle moving in a strong magnetic field. The particle exhibits fast oscillations around the magnetic field lines, which are largely unimportant to measure. Isolating the slow drift motion along and across the field lines is essential in order to efficiently simulate such systems. The concept of *adiabatic invariance* provides the necessary approximately-conserved quantities which allow one to analytically derive reduced Hamiltonian systems for the slow dynamics of charged particle motion. Passage to this reduced system involves averaging over a continuous family of *approximate* symmetries resulting from the adiabatic invariant (discussed in Section “Nearly-periodic Hamiltonian systems”).

In practice, full descriptions of Hamiltonian dynamics in the form of governing equations are challenging to identify from experimental data because the fast-scale oscillations are often underresolved. Moreover, given the full set of governing equations, analytically deriving the reduced-dimension system in the presence of symmetries (or approximate symmetries) becomes infeasible for complex systems. In this article we explore the ability of recent weak-form methods to identify sparse equations for the reduced system in the presence of approximate symmetries, directly from time series data on the Hamiltonian system in question.

Using nearly-periodic systems as a test case, we show in this work that *weak-form equation learning*, specifically the WSINDy algorithm (Weak-form Sparse Identification of Nonlinear Dynamics), which interprets the to-be-discovered dynamics using test functions, provides a framework for directly coarse-graining Hamiltonian systems from observation of only the slow modes. The dictionary learning approach utilized in WSINDy provides a wealth of information on the reduced order system, namely, one can in many cases learn the structure of the entire Hamiltonian from a single noisy trajectory. Moreover, in cases where the Hamiltonian structure is not identified correctly, often the dynamics near the level set of the trajectory are captured very accurately.

The weak form is applied at the level of vector fields. As such, in this work we also provide theoretical results showing that first-order averaged vector fields remain Hamiltonian in the nearly-periodic case. While the all-orders averaging theory of Kruskal^3^ is known to be Hamiltonian, the Hamiltonian structure underlying first-order averaging has never been identified in full generality, thus we provide a self-contained description which, to the best of the authors’ knowledge, constitutes a novel contribution to the theory of averaging.

### Literature review

Significant progress has been made recently in the development of data-driven methods for estimation and identification of Hamiltonian systems and other structured dynamics. These methods include dictionary-based Hamiltonian learning^4^, approximation of Hamiltonian systems and symplectic maps by neural networks^5–7^, and combined dictionary and neural-network based structure preservation in learned dynamics systems^8^. The key difference between these works and ours are (a) we introduce a weak formulation of the discovery problem, which naturally enables discovery from corrupted data, and (b) we demonstrate that the weak form offers direct coarse-graining capabilities, allowing one to identify reduced-order Hamiltonian systems simply by interpreting the dynamics through the action of a suitable class of test functions.

The subject of coarse-graining and reduced-order modeling for Hamiltonian systems has certainly received attention in recent years but this subfield is far from complete. Peng and Mohseni^9^ developed a symplectic reduced-order modeling analogue of proper orthogonal decomposition, which has seen several extensions^10^. From a different perspective, Duruisseaux, Burby, and Tang^11^ introduced a neural-network architecture to specifically handle systems possessing an adiabatic invariant, by which the dynamics may be reduced to an approximate Hamiltonian system of lower dimension. The latter is part of a series of recent works aimed at exploiting near-periodicity to design more efficient means of understanding and simulating dynamics relevant to plasma physics^12–16^. The current work can be seen as a continuation of this series, with the purpose of offering computationally efficient, noise-robust, and interpretable discovery of reduced Hamiltonian systems to complement neural network-based approaches^11^.

Here we focus on the dictionary learning approach to identify reduced Hamiltonian systems. In particular, we employ the WSINDy algorithm, which has its roots in the SINDy algorithm^17^. The weak form has risen to prominence as a way to combat realistic challenges like noisy data and non-smooth dynamics^18–30^. Most relevant to this work, WSINDy has been demonstrated to offer coarse-graining capabilities^31^ in the context of interacting particle systems and homogenization of parabolic PDEs, and more recently in reduced order modeling applications^32^. In a similar vein to the current work, Bramburger, Dylewsky, and Kutz^33^ demonstrate that SINDy may be used to identify reduced dynamics in slow-fast systems. However structure-preservation is not considered, and the method is restricted to systems exhibiting an identifiable separation of scales. Moreover, the method assumes a short timescale characterized by periodic orbits, each with the same period. While this method is complementary to ours, the techniques developed here are very different, and are designed for a more general class of problems. In particular, while the method in^33^ depends on identification of the dominant fast timescale, we demonstrate here that representing the dynamics in weak form is sufficient to coarse-grain the fast scales in addition to combating measurement noise, which can easily hinder the identification of a fast timescale. Our method also allows for fast timescale orbits with variable period.

Compared with POD-based methods^9,10^, which expand the reduced dynamics in terms of a data-driven basis, dictionary learning allows one to learn representations of the reduced dynamics in a basis that easily generalizes as it does not depend on the training dataset. In the context of Hamiltonian equation discovery, the method we study here identifies the Hamiltonian over all of phase space, and can easily be used to explore unseen energy levels. Moreover, structure preservation is easily enforced at the level of the dictionary (see Section “Preliminaries”). On the other end of the spectrum, neural network-based approaches have the capacity to approximate well the underlying dynamics but lack the interpretability and computational efficiency native to dictionary learning. For Hamiltonian equation discovery, neural-networks typically require many trajectories and do not provide global knowledge of the Hamiltonian^11^. However, structure preservation can be enforced in the neural network architecture^5,6,11^.

Ultimately, the main purpose of this work is to demonstrate that the weak form itself has inherent temporal coarse-graining capabilities, which are especially useful in reduced-order Hamiltonian modeling. We note that combinations of weak-form equation learning with other reduced-order modeling paradigms is possible, as exhibited by previous works in other contexts (e.g. POD-based methods^34,35^ and Neural Networks^36^), and we leave these synergies to future work.

### Paper organization

We include preliminary concepts relevant to the study of Hamiltonian coarse-graining in Section “Preliminaries”, namely, an overview of Hamiltonian systems (2.1) with specific attention paid to nearly-periodic Hamiltonian systems (“Nearly-periodic Hamiltonian systems”). The latter is the prototypical class of approximate-symmetries which we use in the current manuscript to investigate weak-form coarse-graining. Section “Hamiltonian structure of first-order averaging theory” contains theoretical results proving that first-order averaging of nearly-periodic Hamiltonian systems at the level of vector fields preserves Hamiltonian structure in the resulting reduced phase space, justifying a search for Hamiltonian coarse-grained models with computations at the level of Hamiltonian vector fields. In Section “WSINDy for hamiltonian systems” we describe how the WSINDy algorithm may be applied to learn a general Hamiltonian system of the form “Hamiltonian systems”. We demonstrate in “WSINDy for Hamiltonian systems with approximate symmetries” that for systems with approximate symmetries, WSINDy can be employed to learn multiple relevant models to describe the system in different regimes. The bulk of our findings is presented in Section “Numerical experiments”, where we quantify the performance of WSINDy applied to four physically-relevant nearly-periodic Hamiltonian systems of varying dimension. We note that Section “Preliminaries” is included to aid in understanding the theoretical results in “Hamiltonian structure of first-order averaging theory”, the weak-form presentation in “WSINDy for hamiltonian systems”, and the examples in “Numerical experiments”.

## Preliminaries

In this Section we review Hamiltonian dynamical systems with specific attention paid to nearly-periodic systems in Section “Nearly-periodic Hamiltonian systems”.

### Hamiltonian systems

Classically, Hamilton’s equations describe the evolution of a point $z=(q,p)$ in phase space $M=\mathbb {R}^{2N}$ along a level set a function $H: M\rightarrow \mathbb {R}$ referred to as the *Hamiltonian*. Hamilton’s original equations are

1 $$\begin{aligned} {\left\{ \begin{array}{ll} \dot{q} = \nabla _p H \\ \dot{p} =- \nabla _q H,\end{array}\right. } \end{aligned}$$

and (*q*, *p*) were originally associated with the position and momentum of a particle (the dot notation denoting the derivative with respect to time). It can readily be seen from Eq. (1) that along any trajectory $t\rightarrow (q(t),p(t))$, *H* is conserved: $\frac{d}{dt}H(q(t),p(t)) = 0.$ By defining the matrix

$$\begin{aligned} \textbf{J}= \begin{pmatrix} 0 &{} {Id}_{\mathbb {R}^N} \\ -{Id}_{\mathbb {R}^N} &{} 0 \end{pmatrix}, \end{aligned}$$

where ${Id}_{\mathbb {R}^N}$ is the identity in $\mathbb {R}^N$, we can equivalently write (1) as $\dot{z} = X_H(z),$ where the *Hamiltonian vector field* $X_H$ is defined

$$\begin{aligned} X_H(z) = \textbf{J}\nabla H(z). \end{aligned}$$

The matrix $\textbf{J}$ is nonsingular, anti-symmetric, and of even dimension, in other words it is *symplectic*. It is this symplectic structure that allows for a significantly more general formulation of Hamiltonian dynamics on arbitrary smooth manifolds using the language of *differential forms*. A differential form $\alpha$ of degree *k* on a smooth manifold *M* is defined as follows. For each point $z\in M$, $\alpha _z$ is an antisymmetric linear mapping from the *k*-fold product tangent space $(T_zM)^k$ to $\mathbb {R}$. In other words, given *k* tangent vectors $\{V_1,\dots ,V_k\}\subset T_zM$, for any odd permutation $\sigma$ on *k* elements we have

$$\begin{aligned} \alpha _z(V_1,\dots ,V_k) = -\alpha _z(V_{\sigma (1)},\dots ,V_{\sigma (k)}) \in \mathbb {R}. \end{aligned}$$

For a comprehensive review of the subject, see textbooks^1,37^ and the exposition^38^ on differential forms in plasma physics. In particular,^1^, Table 2.4-1 contains a reference guide for basic concepts. For the purposes of introducing a widely-applicable weak formulation, we will briefly describe general Hamiltonian systems starting with the following definition.

#### Definition 2.1

(Symplectic manifold) The pair $(M,\Omega )$ is a symplectic manifold if *M* is a smooth manifold and $\Omega$ is a closed, nondegenerate differential 2-form on *M*.

That is, $d\Omega = 0$ (i.e. $\Omega$ is closed, see^1,37,38^ for more details on the exterior derivative *d*) and for every $z\in M$, $\Omega _z$ is a bilinear map on the product tangent space at *z*, $T_zM \times T_zM$, that is anti-symmetric (i.e. $\Omega _z(X,V) = -\Omega _z(V,X)$ for all $X,V\in T_zM$) and non-degenerate (i.e. $\Omega _z(X,V) = 0$ for all $V\in T_zM$ implies $X=0$). Non-degeneracy and anti-symmetry imply that the dimension of *M* must be even. If $\Omega$ is allowed to degenerate, admitting a null space, then $(M,\Omega )$ is referred to as a *presymplectic manifold*, which need not have even dimension. However, throughout we assume that *M* has dimension 2*N* for some $N\ge 1$. We then classify a Hamiltonian system as follows.

#### Definition 2.2

(Hamiltonian system) Let $(M,\Omega )$ be a (2*N*)-dimensional presymplectic manifold and $H: M \rightarrow \mathbb {R}$ a smooth function. The Hamiltonian vector field $X_H$ associated to $(M,\Omega ,H)$ is defined by

2 $$\begin{aligned} \iota _{X_H}\Omega = dH. \end{aligned}$$

The tuple $(M,\Omega ,H,X_H)$ is referred to as a Hamiltonian system.

In (2), *dH* is the differential of *H* and $\iota$ denotes the interior product, which acts on a differential *k*-form $\tau$ and vector field *X* to produce a $(k-1)$-form $\iota _{X}\tau$ defined by $(\iota _{X}\tau )_z(V_1,\dots ,V_{k-1}) = \tau _z(X,V_1,\dots ,V_{k-1})$ for all $V_1,\dots ,V_{k-1}\in T_zM$. Equation (2) will be referred to as *Hamilton’s equations*, representing a generalization of Hamilton’s original equations (1) to arbitrary presymplectic manifolds $(M,\Omega )$. Since $\Omega$ is bilinear, Hamilton’s equations (2) can be written

3 $$\begin{aligned} \Omega (X_H,V) = dH(V), \quad \forall V\in TM. \end{aligned}$$

In this way, $X_H$ is defined implicitly at each point $z\in M$ through the action of elements $\Omega _z(\cdot ,V)$ in $(T_zM)^*$, the dual of the tangent space $T_zM$. This will play a role in the weak formulation below.

### Nearly-periodic Hamiltonian systems

A *nearly-periodic* system is a dynamical system that depends smoothly on a small parameter $\varepsilon$ and is periodic in the limit $\varepsilon \rightarrow 0$. A nearly-periodic *Hamiltonian* system is nearly periodic and is Hamiltonian for all $\varepsilon \ge 0$. In practice, the symplectic form may depend on $\varepsilon$ and degenerate at $\varepsilon =0$, hence why recent developments in nearly-periodic system theory^13,15,16^ work with presymplectic, rather than symplectic Hamiltonian systems (see Examples 3 and 4 below). Nearly-periodic Hamiltonian systems are a prototypical class of dynamics to explore how approximate symmetries may be used to reduce the dimensionality of physical systems. An important example relevant to plasma physics is the dynamics of a charged particle in a strong magnetic field, which is emulated below in Examples 3 and 4.

First, we introduce several definitions. The limiting symmetry in a nearly-periodic system is defined using a *circle action*.

#### Definition 2.3

(Circle Action) A 1-parameter family of diffeomorphisms $\{\Phi _\theta \ :\ \theta \in \mathbb {R}\}$, on a manifold *M* is a circle action if $\Phi _{\theta +2\pi } = \Phi _\theta$, $\Phi _0 = \text {Id}_M$, and $\Phi _{\theta _1+\theta _2} = \Phi _{\theta _1}\circ \Phi _{\theta _2}$.

Change with respect to an underlying vector field on *M* is defined using the *Lie derivative*.

#### Definition 2.4

(Lie Derivative) Let *X* be a vector field on *M* with associated flow map ${\Phi _t}$ such that $\frac{d}{dt}{\Phi _t}(z) = X({\Phi _t}(z))$ for all $z\in M$. The Lie derivative of a tensor field $\tau$ on *M* with respect to *X* is defined by

$$\begin{aligned} {\mathcal {L}}_X\tau (z) = \frac{d}{dt}\Big \vert _{t=0}\Phi _t^*\tau (z) \end{aligned}$$

where $(\cdot )^*$ is the pullback operation (see e.g.^1^, Def. 1.7.16).

For instance, if *f* is a function on *M*, then ${\mathcal {L}}_Xf(z)$ is the directional derivative of *f* at *z* in the direction *X*(*z*). If *V* is a vector field on *M*, then ${\mathcal {L}}_X V$ is the Lie Bracket of *X* and *V*, given by $\partial _X V - \partial _V X$, with $\partial$ denoting the directional derivative. If $\tau$ is a rank-*k* differential form, then the Lie derivative is given by Cartan’s formula,

4 $$\begin{aligned} {\mathcal {L}}_X\tau = \iota _X d\tau + d(\iota _X\tau ). \end{aligned}$$

We will occasionally denote vector fields in differential operator notation, in other words with local coordinates $z=(z_1,\dots ,z_{2N})$ for *M* the vector field $V(z) = (V_1(z),\dots ,V_{2N}(z))$ may be written $V(z) = \sum _{j=1}^{2N} V_j(z)\partial _{z_j}$. As well as providing a compact notation, this emphasizes the action of vector fields on functions through the ${\mathcal {L}}_Vf = df(V)$.

A nearly periodic Hamiltonian System is then defined as follows.

#### Definition 2.5

(Nearly periodic Hamiltonian System) An $\varepsilon$-dependent Hamiltonian system $(M,\Omega _\varepsilon ,H_\varepsilon ,X_\varepsilon )$ is nearly periodic if there exists a function $\omega _0:M\rightarrow \mathbb {R}$ and a circle action $\Phi _\theta$ such that

1. $\Omega _\varepsilon ,H_\varepsilon$ depend smoothly on $\varepsilon$
2. $X_0 = \omega _0R_0$ where $R_0$ is the infinitesimal generator of a circle action $\Phi _\theta$, i.e. $R_0 = \frac{d}{d\theta }\Big \vert _{\theta =0} \Phi _\theta$
3. The limiting angular frequency $\omega _0$ is strictly positive and satisfies ${\mathcal {L}}_{R_0}\omega _0 = 0$.

For a nearly-periodic Hamiltonian system $(M,\Omega _\varepsilon ,H_\varepsilon ,X_\varepsilon )$, smoothness in $\varepsilon$ implies that for sufficiently small $\varepsilon$, $\Omega _\varepsilon$ and $H_\varepsilon$ have formal asymptotic expansions

$$\begin{aligned} H_\varepsilon (z) = H_0(z) +\varepsilon H_1(z) +\varepsilon ^2 H_2(z)+\cdots \\(\Omega _\varepsilon )_z = (\Omega _0)_z +\varepsilon (\Omega _1)_z +\varepsilon ^2 (\Omega _2)_z + \cdots \end{aligned}$$

From Hamilton’s equations (2), we find that the Hamiltonian vector $X_\varepsilon$ has a formal power series $X_\varepsilon = X_0+\varepsilon X_1 + \varepsilon ^2X_2+\cdots$ with coefficient vector fields given by the infinite family of equations

5 $$\begin{aligned} \iota _{X_0}\Omega _0&= dH_0 \end{aligned}$$

6 $$\begin{aligned} \iota _{X_1}\Omega _0+\iota _{X_0}\Omega _1&= dH_1 \end{aligned}$$

7 $$\begin{aligned} \iota _{X_2}\Omega _0+\iota _{X_1}\Omega _1+\iota _{X_0}\Omega _2&= dH_2 \nonumber \\&\vdots \end{aligned}$$

In 1962 M. Kruskal^3^ proved that every nearly periodic system possesses an approximate *U*(1) symmetry given by a unique vector field $R_\varepsilon$ referred to as the *roto-rate*, and defined as follows.

#### Definition 2.6

(Roto-rate) The roto-rate of a nearly periodic vector field $X_\varepsilon$ is a formal power series $R_\varepsilon = R_0 + \varepsilon R_1 +\varepsilon ^2R_2+\cdots$ with vector field coefficients $R_k$ such that $R_0 = \omega _0^{-1}X_0$ and to all orders in $\varepsilon$,

1. $${\mathcal {L}}_{R_\varepsilon }X_\varepsilon = 0$$
2. The integral curves of $R_\varepsilon$ are $2\pi$-periodic.

Kruskal also showed that, in the Hamiltonian case, $R_\varepsilon$ is itself Hamiltonian, with Hamiltonian $\mu _\varepsilon$ known as the *adiabatic invariant*. The adiabatic invariant is defined as a formal power series

$$\begin{aligned} \mu _\varepsilon = \mu _0+\varepsilon \mu _1+\varepsilon ^2\mu _2+\cdots . \end{aligned}$$

Explicit formulas for the first few terms in the series were first found by Burby and Squire^16^. Since $R_\varepsilon$ commutes with $X_\varepsilon$ to all orders in $\varepsilon$ (${\mathcal {L}}_{R_\varepsilon }X_\varepsilon =0$), $\mu _\varepsilon$ is formally conserved by $X_\varepsilon$, hence the notion of $R_\varepsilon$ as an approximate symmetry of $X_\varepsilon$.

### Symmetry reduction in nearly-periodic Hamiltonian systems

In the presence of an exact continuous symmetry $t\rightarrow {\Phi _t}$ of a Hamiltonian system $(M,\Omega ,H,X_H)$, such that ${\mathcal {L}}_RX_H=0$ and $\iota _{R}\Omega = d\mu _*$, where *R* is the infinitesimal generator of ${\Phi _t}$, the phase space $(M,\Omega )$ and dynamics $(H,X_H)$ may be reduced using the Marsden-Weinstein-Meyer construction (see^1^, Chapter 4). At a high level, this proceduring involves (a) restricting to a level set $\Lambda _\mu =\mu _*^{-1}(\mu )$ of the conserved quantity $\mu _*$ associated with ${\Phi _t}$, (b) forming the reduced phase space $M^{\mu }_0$ as an equivalence class of points that lie on the same *R*-orbit, and (c) identifying a suitable symplectic form $\Omega ^{\mu }_0$ on $M^{\mu }_0$. Existence of $\Omega ^{\mu }_0$ on the reduced phase space $M^{\mu }_0$ is at the heart of this result. On the other hand, the reduced Hamiltonian ${\mathcal {H}}^{\mu }_0$ on $M^{\mu }_0$ is simply defined since *H* is, by assumption, constant along *R*-orbits. We note that other analytical methods of reducing Hamiltonian systems do exist, including those related to normal-form theory^39^, which involves successive near-identity coordinate transformations.

In the setting of an approximate symmetry, to derive a reduced Hamiltonian ${\mathcal {H}}^{\mu }_0$ the procedure is similar to Marsden-Weinstein-Meyer, but with substantially more effort for higher orders in $\varepsilon$. For nearly-periodic systems one is guaranteed a reduction in dimension of at least 2, as (1) we restrict to a level set of the adiabatic invariant $\mu _\varepsilon$, and (2) we condense phase space by forming an equivalence class of points that lie on the same orbit of the roto-rate $R_\varepsilon$. However, the reduction in dimension can be greater, as demonstrated below in Section “Numerical experiments”, Example 4.

To connect theory and practice, it is useful to draw parallels between geometric reduction of Hamiltonian systems, such as the Marsden-Weinstein-Meyer construction, and dynamic reduction involving averages and restrictions at the level of vector fields. In the next section we place the latter (dynamic) reduction technique on firmer ground by providing new theoretical results that ensure first-order averaging at the level of $X_\varepsilon$ preserves Hamiltonian structure. These theoretical results complement existing results on nearly periodic systems in^12,16^, and provide motivation for weak-form equation learning from trajectories of nearly-periodic systems. Following is a discussion drawing parallels to classical time-averaging in fast-slow systems.

## Hamiltonian structure of first-order averaging theory

Let $X_\varepsilon = \omega _0\,R_0 + \varepsilon \,X_1 + \dots$ be a nearly-periodic Hamiltonian system on the exact presymplectic manifold $(M,\Omega _\varepsilon )$ with Hamiltonian $H_\varepsilon$ and limiting roto-rate $R_0$. Recall that presymplectic means $\Omega _\varepsilon$ is antisymmetric and closed, $d\Omega _\varepsilon =0$, but possibly degenerate. Exact means there is a 1-form $\vartheta _\varepsilon$ such that $\Omega _\varepsilon = -d\vartheta _\varepsilon$. Hamiltonian means $\iota _{X_\varepsilon }\Omega _\varepsilon = dH_\varepsilon$, which implies the sequence of Eqs. (5)–(7). The flow map for $R_0$ will be denoted $\Phi _\theta ^0$, where $\theta \in \mathbb {R}\text { mod }2\pi$ is the time parameter. Given a tensor $\tau$ on *M* its $R_0$-average will be denoted by

8 $$\begin{aligned} \langle \tau \rangle = (2\pi )^{-1}\int _0^{2\pi }(\Phi _\theta ^0)^*\tau \,d\theta \end{aligned}$$

that is, the time-average of the pullback of $\tau$ by $\Phi ^0_\theta$.

Generic (e.g. possibly non-Hamiltonian) first-order averaging theory approximates the vector field $X_\varepsilon$ with the *first-order average vector field*

$$\begin{aligned} X_\varepsilon ^*= X_0 + \varepsilon \,\langle X_1\rangle . \end{aligned}$$

The first-order average is automatically $R_0$-invariant, ${\mathcal {L}}_{R_0} X_\varepsilon ^*= 0$, meaning the fast phase corresponding to motion along $X_0 = \omega _0\,R_0$ is ignorable after the replacement. We will show that the reduced dynamics defined by $X_\varepsilon ^*$ and obtained by ignoring the fast phase is presymplectic Hamiltonian under the assumptions in the previous paragraph. Although the all-orders averaging theory of Kruskal^3^ is known to be Hamiltonian, the Hamiltonian structure underlying first-order averaging has never been identified in full generality. Since first-order averaging is sufficient for analyzing various important examples, including those considered in this work, a self-contained description here is warranted.

First we recall some useful results related to the adiabatic invariant $\mu _\varepsilon$ from the general theory of nearly-periodic Hamiltonian systems on presymplectic manifolds^16^. Adiabatic invariance means $\mathcal {L}_{X_\varepsilon }\mu _\varepsilon = 0$ in the sense of formal power series. There are general formulas for the first few terms in the series $\mu _\varepsilon$, the simplest being

9 $$\begin{aligned} \mu _0 = \iota _{R_0}\langle \vartheta _0\rangle , \end{aligned}$$

where $\vartheta _0$ denotes the first term in the formal power series expansion for $\vartheta _\varepsilon = \vartheta _0 + \varepsilon \,\vartheta _1 + \dots$. Observe that the exterior derivative of Eq. (9) justifies thinking of the leading-order adiabatic invariant as “energy over frequency,”

10 $$\begin{aligned} d\mu _0 = \iota _{R_0}d\langle \vartheta _0 \rangle = \omega _0^{-1}\iota _{X_0}\langle \Omega _0\rangle = \omega _0^{-1}\,dH_0, \end{aligned}$$

where we have used Eq. (5) to infer $\langle H_0\rangle = H_0$. The $R_0$-average of $\mu _1$ is also simple:

11 $$\begin{aligned} \langle \mu _1\rangle = \iota _{R_0}\langle \vartheta _1\rangle . \end{aligned}$$

In general, some number of the first terms in the series $\mu _\varepsilon$ vanish. Let *k* denote the smallest integer such that $\mu _k$ is not identically 0. Set $\mu ^* = \mu _k$. The condition $\mathcal {L}_{X_\varepsilon }\mu _\varepsilon = 0$ is equivalent to the sequence of equations

12 $$\begin{aligned} \mathcal {L}_{X_0}\mu ^*&= 0 \end{aligned}$$

13 $$\begin{aligned} \mathcal {L}_{X_1}\mu ^* + \mathcal {L}_{X_0}\mu _{k+1}&= 0 \nonumber \\ \vdots \end{aligned}$$

Next we prove a simple technical lemma that establishes structural properties of the first-order averaged system related to its presymplectic Hamiltonian formulation.

### Lemma 1

The first non-zero coefficient $\mu ^*$ in the adiabatic invariant series is constant along solutions of the first-order averaged system $X_\varepsilon ^*$,

14 $$\begin{aligned} \mathcal {L}_{X_\varepsilon ^*}\mu ^* = 0. \end{aligned}$$

In addition, the first-order averaged system satisfies the Hamilton-like equation

15 $$\begin{aligned} \iota _{X_\varepsilon ^*}\Omega _0=dH_0 + \varepsilon \,d\langle H_1\rangle - \varepsilon \,\omega _0\,d\langle \mu _1\rangle . \end{aligned}$$

### Proof

The conservation law (14) follows immediately from (12) and the $R_0$-average of (13). (Notice that $\langle \mathcal {L}_{X_0}\mu _1\rangle = \omega _0\,\langle \mathcal {L}_{R_0}\mu _1\rangle = 0$ and $\langle \mu ^*\rangle = \mu ^*$.) As for (15), it follows directly from the $R_0$-average of (6) and the identity

16 $$\begin{aligned} \iota _{X_0}\langle \Omega _1\rangle = -\omega _0\langle \iota _{R_0}d\vartheta _1\rangle = \omega _0\,d(\iota _{R_0}\langle \vartheta _1\rangle ) = \omega _0\,d\langle \mu _1\rangle , \end{aligned}$$

which represents an application of Cartan’s formula (4) and (11). $\square$

Because $\mu ^*$ is automatically conserved by the first-order averaged system we can study $X_\varepsilon ^*$ one level set of $\mu ^*$ at a time. Let $\Lambda _\mu = (\mu ^*)^{-1}\{\mu \}$ be a regular level set for $\mu ^*$ with regular value $\mu$. By Lemma 1$X_\varepsilon ^*$ restricts to a vector field $X_{\mu }^*$ on $\Lambda _\mu$. It turns out that $X_{\mu }^*$ satisfies a genuine Hamilton equation, even though $X_\varepsilon ^*$ is not Hamiltonian in general due to the inexact 1-form $\omega _0\,d\langle \mu _1\rangle$ in Eq. (15).

### Lemma 2

Let $i_\mu :\Lambda _\mu \rightarrow M$ denote the canonical inclusion map. Define the closed 2-form $\Omega _{\mu }^*=i_\mu ^*\Omega _0$ and the function ${\mathcal {H}}_{\mu }^*= i_\mu ^*(H_0 + \varepsilon \,\langle H_1\rangle - \varepsilon \,\omega _0\langle \mu _1\rangle )$. The restricted first-order averaged system $X_{\mu }^*$ is $\Omega _{\mu }^*$-Hamiltonian with Hamiltonian ${\mathcal {H}}_{\mu }^*$,

17 $$\begin{aligned} \iota _{X_{\mu }^*}\Omega _{\mu }^*= d{\mathcal {H}}_{\mu }^*. \end{aligned}$$

### Proof

Pulling back Eq. (15) along $i_\mu$ implies

$$\begin{aligned} \iota _{X_{\mu }^*}\Omega _{\mu }^*= di_\mu ^*\big (H_0 + \varepsilon \,\langle H_1\rangle \big ) - \varepsilon \,i_\mu ^*\big (\omega _0\,d\langle \mu _1\rangle \big ). \end{aligned}$$

So the proof merely requires showing $i_\mu ^*\big (\omega _0\,d\langle \mu _1\rangle \big ) = di_\mu ^*(\omega _0\,\langle \mu _1\rangle )$. We consider three cases separately.

*case *1: $\mu ^* = \mu _0$. In this case the energy-period relation (10) implies $d\omega _0 \wedge d\mu ^* = d\omega _0\wedge d\mu _0= ddH_0 = 0$. Thus, $d\omega _0 = \lambda \,d\mu ^*$, for some function $\lambda$. Pulling back along $i_\mu$ then implies $di_\mu ^*\omega _0 = 0$, which says that $i_\mu ^*\omega _0$ is constant. Since the exterior derivative is $\mathbb {R}$-linear, we therefore find

$$\begin{aligned} i_\mu ^*\big (\omega _0\,d\langle \mu _1\rangle \big ) = (i_\mu ^*\omega _0)di_\mu ^*\langle \mu _1\rangle = d\big ((i_\mu ^*\omega _0)i_\mu ^*\langle \mu _1\rangle \big ) = di_\mu ^*(\omega _0\,\langle \mu _1\rangle ), \end{aligned}$$

as desired.

*case *2: $\mu ^* = \mu _1$. In this case Eq. (12) implies $\mu ^* = \mu _1 = \langle \mu _1\rangle$. Therefore

$$\begin{aligned} i_\mu ^*\big (\omega _0\,d\langle \mu _1\rangle \big ) = (i_\mu ^*\omega _0)di_\mu ^*\langle \mu _1\rangle = (i_\mu ^*\omega _0)di_\mu ^*\mu ^* = 0, \end{aligned}$$

as desired.

*case *3: $\mu ^* = \mu _k$, $k >1$. In this case both $\mu _0$ and $\mu _1$ vanish identically. Therefore

$$\begin{aligned} i_\mu ^*\big (\omega _0\,d\langle \mu _1\rangle \big ) = i_\mu ^*\big (\omega _0\,d0\big )=0, \end{aligned}$$

as desired. $$\square$$

We are finally in position to establish our main claim: after “forgetting” the fast phase in the first-order averaged system, the resulting slow dynamics are presymplectic Hamiltonian. The result follows from Lemma 2 and a variant of the Marsden-Weinstein symplectic quotient technique.

### Theorem 1

Let $M^{\mu }_0= \Lambda _\mu /U(1)$ denote the space of $R_\mu$-orbits in $\Lambda _\mu$. Assume it is a manifold. Let $\pi :\Lambda _\mu \rightarrow M^{\mu }_0$ denote the corresponding quotient map. On $M^{\mu }_0$, there is a unique vector field $X^{\mu }_0$, a unique closed 2-form $\Omega ^{\mu }_0$, and a unique function ${\mathcal {H}}^{\mu }_0$ such that

18 $$\begin{aligned} T\pi \circ X_{\mu }^*= X^{\mu }_0\circ \pi , \quad \pi ^*\Omega ^{\mu }_0= \Omega _{\mu }^*,\quad \pi ^*{\mathcal {H}}^{\mu }_0= {\mathcal {H}}_{\mu }^*. \end{aligned}$$

Moreover, $X^{\mu }_0$ is $\Omega ^{\mu }_0$-Hamiltonian with Hamiltonian function ${\mathcal {H}}^{\mu }_0$,

19 $$\begin{aligned} \iota _{X^{\mu }_0}\Omega ^{\mu }_0= d{\mathcal {H}}^{\mu }_0. \end{aligned}$$

### Proof

By construction, the first-order averaged system commutes with $R_0$, ${\mathcal {L}}_{R_0}X_\varepsilon ^*= 0$. By Eqs. (12) and (14) both $R_0$ and $X_\varepsilon ^*$ are tangent to $\Lambda _\mu$. Therefore ${\mathcal {L}}_{R_\mu }X_{\mu }^*= 0$, which says that the $X_{\mu }^*$-flow maps $R_\mu$-orbits into $R_\mu$-orbits. It follows that there is a unique vector field $X^{\mu }_0$ on orbit space $M^{\mu }_0$ that is $\pi$-related to $X_{\mu }^*$.

In general, the interior product of $R_\mu$ with $\Omega _{\mu }^*$ is given by $\iota _{R_\mu }\Omega _{\mu }^*= i_\mu ^*(\iota _{R_0}\Omega _0) = di_\mu ^*\mu _0$. When $\mu ^* = \mu _0$, $i_\mu ^*\mu _0$ is constant and $\iota _{R_\mu }\Omega _{\mu }^*= 0$. When $\mu ^* = \mu _k$, $k>0$, $\mu _0 =0$ and, again, $\iota _{R_\mu }\Omega _{\mu }^*= 0$. So $\iota _{R_\mu }\Omega _{\mu }^*= 0$ in general. It follows that there is a unique 2-form $\Omega ^{\mu }_0$ on $M^{\mu }_0$ such that $\pi ^*\Omega ^{\mu }_0= \Omega _{\mu }^*$. It is straightforward to show that $d\Omega ^{\mu }_0= 0$.

Since $\mathcal {L}_{R_\mu }{\mathcal {H}}_{\mu }^*=0$ there is a unique function ${\mathcal {H}}^{\mu }_0$ such that $\pi ^*{\mathcal {H}}^{\mu }_0= {\mathcal {H}}_{\mu }^*$. Overall, we have just established existence of the desired objects on $M^{\mu }_0$ that satisfy Eq. (18). Establishing the Hamilton equation (19) is now simple. Notice that

$$\begin{aligned} \iota _{X_{\mu }^*}\Omega _{\mu }^*= \iota _{X_{\mu }^*}\pi ^*\Omega ^{\mu }_0= \pi ^*(\iota _{X^{\mu }_0}\Omega ^{\mu }_0) = \pi ^*d{\mathcal {H}}^{\mu }_0, \end{aligned}$$

which says that the 1-form $\pi ^*(\iota _{X^{\mu }_0}\Omega ^{\mu }_0-d{\mathcal {H}}^{\mu }_0)$ on $\Lambda _\mu$ vanishes. But because $\pi$ is a surjective submersion $\pi ^*$ is injective. It follows that $\iota _{X^{\mu }_0}\Omega ^{\mu }_0-d{\mathcal {H}}^{\mu }_0= 0$, as claimed. $\square$

From Eqs. (15), (17), and (19), we can identify two methods of obtaining the leading-order reduced Hamiltonian system $(M^{\mu }_0,\Omega ^{\mu }_0,{\mathcal {H}}^{\mu }_0,X^{\mu }_0)$. To clarify terminology, we refer to $(M^{\mu }_0,\Omega ^{\mu }_0,{\mathcal {H}}^{\mu }_0,X^{\mu }_0)$ as the “leading-order reduced” system since it is obtained from utilizing the roto-rate $R_\varepsilon$ and adiabatic invariant $\mu _\varepsilon$ each to leading-order (while $X_\varepsilon ^*$ is the “first-order averaged” vector field since it is obtained from averaging $X_\varepsilon$ to first order, $X_\varepsilon ^*= \langle X_0+\varepsilon X_1\rangle$). Higher-order reduced systems can be obtained in a similar manner when given access to higher-order terms in $R_\varepsilon$ and $\mu _\varepsilon$, however the order-by-order Hamiltonian structure is as-of-yet undetermined, and we reserve it for a future work. We will differentiate the two methods of obtaining $(M^{\mu }_0,\Omega ^{\mu }_0,{\mathcal {H}}^{\mu }_0,X^{\mu }_0)$ as *geometric reduction* and *dynamic reduction*:

Method I: *Geometric reduction*.
  - Average $(H_\varepsilon ,\Omega _\varepsilon )$ around $R_0$ to first-order in accordance with Hamilton’s equations (5)–(7), yielding $(H_\varepsilon ^*,\Omega _\varepsilon ^*)$
  - Restrict $(H_\varepsilon ^*,\Omega _\varepsilon ^*)$ to the level set $\Lambda _\mu = \mu _*^{-1}\{\mu \}$ to obtain ${\mathcal {H}}_{\mu }^*= i_\mu ^*(H_\varepsilon ^*-\varepsilon \omega _0\langle \mu _1\rangle )$ and $\Omega _{\mu }^*= i_\mu ^*\Omega$, having adjusted the energy $H_\varepsilon ^*$ according to the ${\mathcal {O}}(\varepsilon )$ adiabatic invariant $\mu _1$
  - Quotient out $\Lambda _\mu$ by orbits of $R_{\mu }= i_\mu ^*R_0$ to get the reduced phase space $M^{\mu }_0$, reducing $({\mathcal {H}}_{\mu }^*,\Omega _{\mu }^*)$ through $R_{\mu }$-invariance to $({\mathcal {H}}^{\mu }_0,\Omega ^{\mu }_0)$
  - Obtain the reduced Hamiltonian vector field $X^{\mu }_0$ on $M^{\mu }_0$ using Hamilton’s equations applied to $({\mathcal {H}}^{\mu }_0,\Omega ^{\mu }_0)$
Method II: *Dynamic reduction*.
  - Obtain $X_\varepsilon ^*$ by averaging the first-order vector field $X_0+\varepsilon X_1$ around the flow of $R_0$
  - Notice that for regular values of $\mu _*$, $X_\varepsilon ^*$ is tangent to $\Lambda _\mu = \mu _*^{-1}\{\mu \}$, hence obtain $X_{\mu }^*$ as the restriction of $X_\varepsilon ^*$ to $\Lambda _\mu$
  - Represent the quotient space $M^{\mu }_0$ as a section from $\Lambda _\mu$ with each pointcorresponding to a unique $R_{\mu }$-orbit lying in $\Lambda _\mu$
  - $X_{\mu }^*$ restricted to this section equals $X^{\mu }_0$ and is Hamiltonian according to (19)

Geometric reduction is more robust and allows for systematic exploration of higher-order reductions in $\varepsilon$. On the other hand, dynamic reduction merely involves averaging around the flow of the limiting roto-rate $R_0$ and observing the resulting system on the reduced submanifold $M^{\mu }_0$. In physical systems, it can be assumed that the system is already close to this manifold, hence some method of averaging observations is all that is needed to observe the Hamiltonian structure of the system to first order. This is exactly the proporty exploited by the weak formulation in the current work. The weak form emulates the roto-rate averaging necessary to reveal Hamiltonian structure in the reduced dynamics.

### Connection to classical time-averaging

Classical time-averaging has been employed to treat nonautonomous dynamical systems of the form $\dot{z}(t) = \varepsilon F(z(t),t)$ such that *F* is highly oscillatory in its second argument compared to the timescale dictated by $\varepsilon$ (for a review of the subject see the textbook^40^). It can be shown that if *F* is *T*-periodic in its second argument, then the time-averaged vector field

$$\begin{aligned} \overline{F}(z):= \frac{1}{T}\int _0^{T} F(z,t)\,dt \end{aligned}$$

produces *autonomous* dynamics $\dot{\overline{z}} = \overline{F}(\overline{z})$ that remain $\varepsilon$-close to *z* over ${\mathcal {O}}(\varepsilon ^{-1})$ time windows^40^, Ch.2 when $\overline{z}(0) = z(0)$.

We can now make some connections between the theoretical results of the previous section and classical time-averaging of fast-slow systems. Consider the setting of Examples 1 and 2 of Section “Numerical experiments” with dynamics in $\mathbb {R}^{2N}$ that can be partitioned into slow and fast modes, $z = (z_s,z_f)\in \mathbb {R}^{2(N-1)}\times \mathbb {R}^{2}$, with Hamiltonian $H_\varepsilon$ of the form

$$\begin{aligned} H_\varepsilon (z) = H_0(z_f) + \varepsilon H_1(z_s,z_f). \end{aligned}$$

The first-order averaged Hamiltonian then takes the form, for some $\widetilde{H}:\mathbb {R}^{2(N-1)}\times \mathbb {R}\rightarrow \mathbb {R}$,

$$\begin{aligned} H_\varepsilon ^*(z) = H_0(z_f) + \varepsilon \widetilde{H}(z_s,\mu _0(z_f)) \end{aligned}$$

where $\mu _*=\mu _0$ is the leading-order adiabatic invariant (see Eq. ). When the symplectic form $\Omega _\varepsilon$ is independent of $\varepsilon$ (i.e. $\Omega _\varepsilon = \Omega _0$), the first-order averaged dynamics $X_\varepsilon ^*$ are already Hamiltonian (see (15), (16)) given by $\iota _{X_\varepsilon ^*} \Omega _0 = dH_\varepsilon ^*$. If we further have $\iota _{V_s}\iota _{V_f}\Omega _0=0$ for any pair of vector fields $V_s$, $V_f$ tangent to the slow and fast submanifolds, respectively, then the dynamics $\dot{\tilde{z}} = X_\varepsilon ^*(\tilde{z})$ consist of slow and fast subsystems $\tilde{z}_s$ and $\tilde{z}_f$ with $\tilde{z}_s$ *decoupled from* $\tilde{z}_s$. Explicitly, the dynamics are given by

20 $$\begin{aligned} {\left\{ \begin{array}{ll} \dot{\tilde{z}}_s = \varepsilon \textbf{J}_s\nabla _{z_s} \widetilde{H}(\tilde{z}_s,\mu _0(\tilde{z}_f)) \\ \dot{\tilde{z}}_f = \textbf{J}_f \Big (\nabla _{z_f}H_0(\tilde{z}_f)\ +\ \varepsilon \partial _2 \widetilde{H}(\tilde{z}_s,\mu _0(\tilde{z}_f))\nabla _{z_f} \mu _0(\tilde{z}_f)\Big ) \end{array}\right. } \end{aligned}$$

with $\partial _2$ denoting differentiation with respect to the second argument and symplectic matrices $\textbf{J}_s$, $\textbf{J}_f$ derived from $\Omega _0$ for the slow and fast subsystems. To see that $\tilde{z}_s$ is now decoupled from $\tilde{z}_f$, notice that for any trajectory $\tilde{z}=(\tilde{z_s},\tilde{z}_f)$ of (20) it holds that

$$\begin{aligned} \frac{d}{dt}\mu _0(\tilde{z}(t))\ =\ \nabla _{z_f}\mu _0(\tilde{z}_f)\cdot \textbf{J}_f \nabla _{z_f}H_0(\tilde{z}_f)\ +\ \left[ \nabla _{z_f}\mu _0(\tilde{z}_f)\cdot \textbf{J}_f \nabla _{z_f} \mu _0(\tilde{z}_f)\right] \left( 1+\varepsilon \partial _2 \widetilde{H}(\tilde{z}_s,\mu _0(\tilde{z}_f))\right) \ =\ 0 \end{aligned}$$

where the first term is zero because $\mu _0$ is conserved by the flow of $X_0$, or

$$\begin{aligned} \nabla _{z_f}\mu _0(\tilde{z}_f)\cdot \textbf{J}_f \nabla _{z_f}H_0(\tilde{z}_f) = \nabla _{z_f}\mu _0(\tilde{z}_f)\cdot X_0(\tilde{z}) = {\mathcal {L}}_{X_0}\mu _0 (\tilde{z}) = 0,\end{aligned}$$

and the second term is zero because $\textbf{J}_f$ is symplectic. From the dependence of $\widetilde{H}$ on $\mu _0$, conservation of $\mu _0$ along this reduced flow implies that the slow system $\tilde{z}_s$ has decoupled from the fast system $\tilde{z}_f$. Reducing phase space according to $M\rightarrow \Lambda _\mu \rightarrow M^{\mu }_0$ is then equivalent to restriction to the slow variables $\tilde{z}_s$, and the reduced Hamiltonian is given by ${\mathcal {H}}^{\mu }_0= \varepsilon \widetilde{H}(z_s,\mu )$.

Let us now treat this problem from the perspective of classical time-averaging. The slow $z_s$ dynamics can be written as the nonautonomous system

21 $$\begin{aligned} \dot{z}_s = \varepsilon F(z_s,t), \end{aligned}$$

suppressing explicit dependence on $z_f$ through the second argument of *F*. Since it holds that $z_f(t) = (\Phi ^0_t z(0))_f+{\mathcal {O}}(\varepsilon )$, for $\Phi ^0_t$ the flow-map of the roto-rate $R_0$, we see that *F* is ${\mathcal {O}}(\varepsilon )$ close to being $2\pi$-periodic in its second argument. By classical time-averaging theory, the time-averaged system

$$\begin{aligned} \dot{\overline{z}} = \varepsilon \overline{F}(\overline{z}), \qquad \overline{F}(z):= \frac{1}{2\pi }\int _0^{2\pi } F(z,t)\,dt \end{aligned}$$

then stays ${\mathcal {O}}(\varepsilon )$ close to $z_s$, and hence to the slow portion of the averaged system (20), on timescales of ${\mathcal {O}}(\varepsilon ^{-1})$. Thus, in this scenario, the techniques of classical time-averaging and first-order averaging of nearly-periodic systems agree to leading order.

While classical time-averaging is very useful for systems (21) with *F* exactly $2\pi$-periodic in its second argument, this is the extent of its utility, and its application to nearly-periodic systems is severely limited. The fast mode $z_f$ is only $2\pi$-periodic when $\varepsilon =0$, such that for any $\varepsilon >0$, the slow and fast systems are coupled, with the dynamics for $z_s$ not falling into the class (21) with *F* periodic in *t*. Deriving the correct analytical formulas for reductions of nearly-periodic systems is a more subtle and labor-intensive task (if not intractable), especially if a Hamiltonian structure is to be preserved (as outlined in the previous section), but significant progress has been made in this direction^12,15,16^.

## WSINDy for hamiltonian systems

The aim of the current work is to demonstrate that weak-form equation learning is highly effective at learning reduced Hamiltonian dynamics for systems exhibiting approximate symmetries, and can be used in concert with analytical techniques. Given the intricacies of analytically reducing a nearly periodic system by means of the roto-rate $R_\varepsilon$, it is surprising that the weak form recovers the same reduced dynamics from discrete-time trajectory data. This requires a general weak-form framework for Hamiltonian dynamics, which we now povide as an extension of WSINDy.

WSINDy is a sparse regression-based algorithm capable of identifying governing differential equations from corrupted sample trajectories^23^. In particular, WSINDy identifies differential equations in a suitable weak formulation through the action of test functions. In this section we describe how WSINDy may be used to identify the Hamiltonian *H* defining a Hamiltonian system using discrete-time observations of the flow of $X_H$. For the remainder of this article we will work in $M=\mathbb {R}^{2N}$, although many of the concepts that follow have direct extensions to general manifolds. We will also assume that the symplectic form $\Omega$ is known, and leave identification of $\Omega$ and extension to general manifolds to future work.

We return to Hamilton’s equations as written in (3), with Hamiltonian system $(M=\mathbb {R}^{2N},\Omega ,H,X_H)$. If $\Omega$ is symplectic, we can associate $\Omega$ with a quadratic form and use the Euclidean inner product (dot product) to write

$$\begin{aligned} \Omega _z(X_H,V) = (\textbf{J}(z)^{-1}X_H)\cdot V, \quad dH(V) = \nabla H\cdot V \end{aligned}$$

where $\textbf{J}(z)$ is a symplectic matrix associated with $\Omega _z$ for each $z\in \mathbb {R}^{2N}$. Suppressing the *z*-dependence, Hamilton’s equations take the form

22 $$\begin{aligned} V\cdot X_H = V\cdot \textbf{J}\nabla H, \quad \forall V\in T\mathbb {R}^{2N}. \end{aligned}$$

We can define a weaker version of (22) (i.e. requiring less regularity of the resulting integral curves of $X_H$) by considering a trajectory $z:[0,T]\rightarrow \mathbb {R}^{2N}$ satisfying

$$\begin{aligned} \dot{z} = X_H(z):=\textbf{J}(z) \nabla H(z) \end{aligned}$$

and using integration by parts in time against a smooth time-dependent vector field *V*(*z*, *t*) satisfying $V(z(0),0) = V(z(T),T) = 0$:

23 $$\begin{aligned} -\int _0^T \left( \frac{d}{dt}{V}(z(t),t)\right) \cdot z(t)\,dt = \int _0^T V(z(t),t)\cdot \textbf{J}(z(t)) \nabla H(z(t))\,dt. \end{aligned}$$

If *V* has no explicit *z*-dependence, the left-hand side can be evaluated without differentiating *z*, leading to

24 $$\begin{aligned} -\int _0^T \dot{V}(t)\cdot z(t)\,dt = \int _0^T V(t)\cdot \textbf{J}(z(t)) \nabla H(z(t))\,dt. \end{aligned}$$

On the other hand, if *V* depends on *z*, we can use the dynamics $\dot{z} = X_H(z)$ and rearrange terms to get

$$\begin{aligned} -\int _0^T\partial _tV(z(t),t)\cdot z(t)\,dt = \int _0^T\left( V(z(t),t) + z\cdot \nabla V(z(t),t)\right) \cdot \textbf{J}(z(t)) \nabla H(z(t))\,dt. \end{aligned}$$

In either case, the phase space variables *z* are not differentiated. This is crucial to accurate identification of *H* from time series data that is corrupted from measurement noise. We will show here that this weak formulation also serves to filter out intrinsic dynamics that occur on a faster timescale. We restrict ourselves to the simpler case (24) in this work and leave full exploration of (23) with general test vector fields $V = V(z,t)$ to future research.

To identify *H* from data, we consider noisy evaluations $\textbf{Z}= z(\textbf{t})+\eta$ where $t\rightarrow z(t)$ is a trajectory from $(\mathbb {R}^{2N},\Omega ,H,X_H)$, $\textbf{t}= (t_0,\dots ,t_{m+1})$ are the timepoints, and $\eta$ represents i.i.d. mean-zero measurement noise with variance $\sigma ^2<\infty$. We approximate *H* by expanding in terms of a chosen library $\mathbb {H}=(H_1,\dots ,H_J)$ of *J* trial Hamiltonian functions,

$$\begin{aligned} \widehat{H} = \sum _{j=1}^J{\widehat{\textbf{w}}}_jH_j:=\mathbb {H}{\widehat{\textbf{w}}}\end{aligned}$$

and we make the assumption that ${\widehat{\textbf{w}}}$ is sparse. To solve for ${\widehat{\textbf{w}}}$, we first discretize (24) using *K* test vector fields $\mathbb {V}= (V_1,\dots ,V_K)$ to arrive at the linear system $(\textbf{b},\textbf{G})\in \mathbb {R}^K\times \mathbb {R}^{K\times J}$ defined by

25 $$\begin{aligned} \textbf{b}_k = -\left\langle \dot{V}_k, \textbf{Z}\right\rangle _{\textbf{t}}, \quad \textbf{G}_{kj} = \left\langle V_k, \textbf{J}(\textbf{Z})\nabla H_j(\textbf{Z})\right\rangle _{\textbf{t}}. \end{aligned}$$

Here, $\left\langle \cdot ,\cdot \right\rangle _\textbf{t}$ defines a discrete inner product on $\mathbb {R}^{2N}$-valued functions of time using $\textbf{t}$ as quadrature nodes. For simplicity and previously demonstrated benefits^22,23^, we use the trapezoidal rule throughout, so that

$$\begin{aligned} \left\langle V,X\right\rangle _\textbf{t}:= \sum _{i=0}^{m} \frac{\Delta t_i}{2}\left( {V_i}\cdot X_i+{V_{i+1}}\cdot X_{i+1}\right) \end{aligned}$$

where $V_i := V(t_i)$ and $\Delta t_i := t_{i+1}-t_i$. For compactly supported *V* (or *X*) in time, this reduces to

$$\begin{aligned} \left\langle V,X\right\rangle _\textbf{t}= \sum _{i=1}^{m} \left( \frac{\Delta t_i+\Delta t_{i-1}}{2}\right) {V_i}\cdot X_i. \end{aligned}$$

In this work, we use the convolutional approach as in^22^, that is, we fix a reference test function $\phi \in C^\infty _c(\mathbb {R})$ supported on $[-T_\phi /2,T_\phi /2]$ for some radius $T_\phi$, and we set the test vector fields to

$$\begin{aligned} V_k(z,t) = \phi (t-t_k)\sum _{j=1}^{2N} \partial _{z_j}, \qquad 1\le k\le K \end{aligned}$$

for a fixed set of *query timepoints* ${\mathcal {Q}}:= \{t_k\}_{k=1}^K$. With $(\textbf{G},\textbf{b})$ defined in (25) under this quadrature, we then solve the sparse regression problem

26 $$\begin{aligned} {\widehat{\textbf{w}}}= \mathop {\textrm{argmin}}\limits _\textbf{w}\Vert \textbf{G}\textbf{w}-\textbf{b}\Vert _2^2+\lambda ^2\Vert \textbf{w}\Vert _0, \end{aligned}$$

where in this work we use the MSTLS algorithm to solve (26) (modified Sequential Thresholding Least Squares^22^), which uses sequential thresholding on the combined term $\Vert \textbf{G}_j\textbf{w}_j\Vert _2/\Vert \textbf{b}\Vert _2$ and coefficient magnitudes $|\textbf{w}_j|$. In addition, MSTLS performs a grid search for a suitable value of $\lambda$ (see^22^ for more details and^17^ for the original STLS algorithm). More information on MSTLS is provided in Appendix S1.

Data from multiple trajectories can easily be incorporated to improve the recovery process. When *L* trajectories $\textbf{Z}= (\textbf{Z}^{(1)},\dots ,\textbf{Z}^{(L)})$ are available, with samples $\textbf{Z}^{(\ell )} = z^{(\ell )}(\textbf{t}^{(\ell )})+\varepsilon$, the linear system $(\textbf{G},\textbf{b})$ is formed by vertically concatenating the linear systems from each trajectory, $\textbf{G}= [(\textbf{G}^{(1)})^T\ |\ \cdots \ |\ (\textbf{G}^{(L)})^T ]^T$, $\textbf{b}= [(\textbf{b}^{(1)})^T\ |\ \cdots \ |\ (\textbf{b}^{(L)})^T ]^T$. Note that the test vector fields in this case need not be the same for each trajectory. In the current work however, we focus on demonstrating recovery of a suitable reduced Hamiltonian ${\mathcal {H}}^{\mu }_0$ using only a single trajectory.

### WSINDy for Hamiltonian systems with approximate symmetries

**Figure 1:**
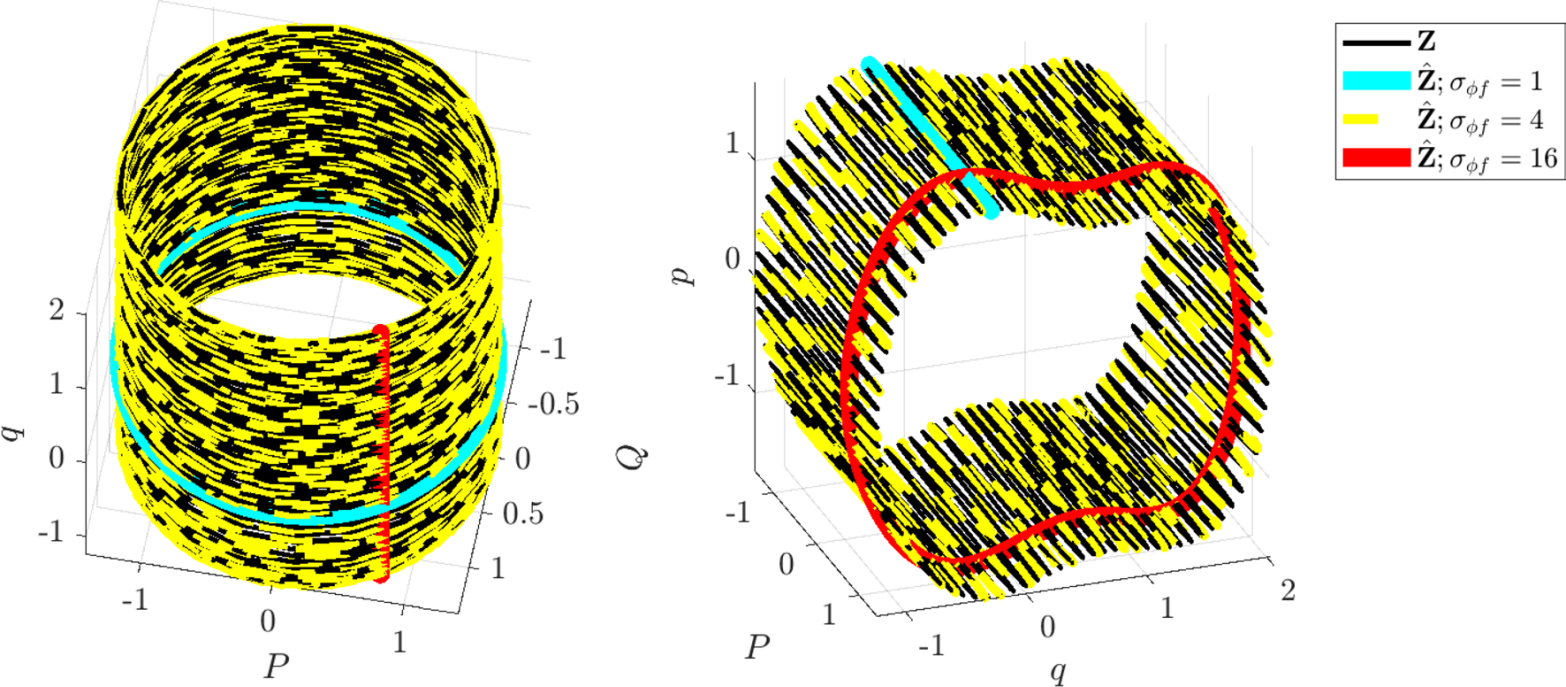
Visualizing multi-model inference. Trajectories $\widehat{\textbf{Z}}$ (cyan, yellow, red) learned using WSINDy applied to data $\textbf{Z}$ (black) from the nearly-periodic system in Example 1 (Eq. 29) with $\varepsilon =0.01$, $1\%$ added Gaussian white noise, and *z*(0)-index 7 (see Figure S2 in the Supplemental Information), for $\sigma _{\phi f}\in \{1,4,16\}$ (Eq. 27). Left and right: (*Q*, *P*, *q*) and (*P*, *q*, *p*) subdomains of phase space $(Q,P,q,p)\in \mathbb {R}^4$. As $\sigma _{\phi f}$ is increased, the learned Hamiltonian transitions from $H_0$ (cyan), to $H_\varepsilon$ (yellow), to ${\mathcal {H}}^{\mu }_0$ (red), identifying the limiting roto-rate, the full system, and the leading-order reduced dynamics.

An intriguing aspect of Hamiltonian systems that exhibit approximate symmetries, from a computational standpoint, is that they typically admit a hierarchy of models which can be used to efficiently express the dynamics in different regimes. These consist of the dynamics of the limiting roto-rate $R_0$, the full Hamiltonian vector field $X_\varepsilon$, and reduced dynamics for each order in $\varepsilon$ obtained from averaging and restricting $X_\varepsilon$ according to the methodology in Section “Hamiltonian structure of first-order averaging theory”. Theorem  1 indicates that the leading-order reduced vector-field $X^{\mu }_0$ is Hamiltonian, and we conjecture that similar conditions exist for higher-order-averaged systems to remain Hamiltonian, justifying a search for Hamiltonian structure in coarse-grained models of $X_\varepsilon$. While the focus of this article is to demonstrate that weak-form methods can be used to identify a coarse-grained Hamiltonian system that agrees with averaging theory, in this Subsection we demonstrate a more general property, that when given access to all state variables, WSINDy allows access to multiple models from the hierarchy associated with nearly-periodic Hamiltonian systems, simply by varying the test function radius $T_\phi$. That is, this Subsection provides justification for a complete analysis of this multi-model inference capability, which will be provided in a future work.

**Figure 2:**
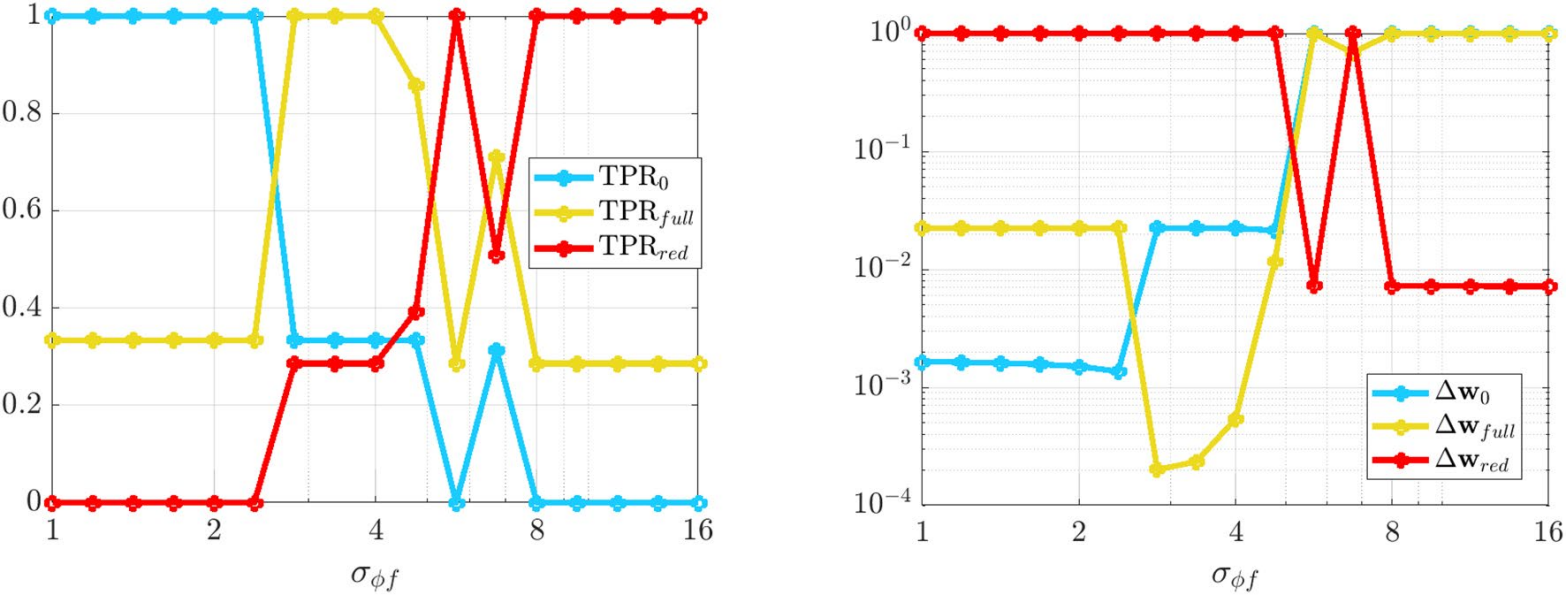
Quantifying multi-model inference. Transitions in the WSINDy output from $H_0$ (blue), to $H_\varepsilon$ (yellow), to ${\mathcal {H}}^{\mu }_0$ (red) as $\sigma _{\phi f}$ (Eq. 27) is increased using data as in Fig. 1. Results are averaged over 100 instantiations of $1\%$ noise. The left and right plots show the TPR values (Eq. 47, a TPR of 1 indicates identification of the true model) and coefficient errors $\Delta \textbf{w}_{(*)}:=\left\| {{\widehat{\textbf{w}}}-\textbf{w}_{(*)}} \right\| _2/\left\| {\textbf{w}_{(*)}} \right\| _2$ for coefficients $\textbf{w}_{(*)}$ defining the Hamiltonians $H_0$, $H_\varepsilon$, and ${\mathcal {H}}^{\mu }_0$, associated with subscripts “0”, “full”, and “red” respectively.

The advantages of weak-form coarse-graining can be clearly observed by examining the performance of WSINDy as a function of the *weak-form scale selector*

27 $$\begin{aligned} \sigma _{\phi f} = \frac{T_\phi }{T_f} \end{aligned}$$

where $T_\phi$ is the support length (in time) of the test function $\phi$ and $T_f$ is the dominant period of the fast scale. In words, $\sigma _{\phi f}$ is the number of fast periods that occur in one integration of the dynamics against $\phi$. Figures 1 and 2 depict the performance of WSINDy as $\sigma _{\phi f}$ is varied from 1 to 16 (i.e. $T_\phi$ is varied from $T_f$ to $16T_f$) when applied to the full 4-dimensional nearly-periodic Hamiltonian system (29) in Example 1. Here the library $\mathbb {H}$ contains only the 7 terms necessary to represent all three relevant models, that is, the leading order dynamics given by $H_0 = \frac{1}{2}(Q^2+P^2)$, the full dynamics given by $H_\varepsilon$ (Eq. 28), and the reduced Hamiltonian ${\mathcal {H}}^{\mu }_0$ (Eq. 31). Figure 2 demonstrates that simply by varying the support of the test function, we gain access to all three models. The added 1% noise affects the transition between models (Fig. 2, left; with lower noise requiring larger $\sigma _{\phi f}$ to obtain the reduced model) as well as the recovered coefficient accuracy (Fig. 2, right), which is less that $1\%$ for each model in its range of validity (i.e. when $\text {TPR}=1$, see Eq. (47)). Figure 1 provides visualizations of the learned dynamics $\widehat{\textbf{Z}}$ in phase space, demonstrating that the dynamics can be approximately described as two commutative flows given by the coarse-grained (red) and roto-rate (cyan) curves (see Fig. S4 in the Supplemental Information for related time series plots). Hence, from a single dataset, the weak form allows access to three models that are fundamental to understanding a nearly-periodic Hamiltonian system.

For the remainder of the article, we focus solely on the ability of WSINDy to identify a suitable coarse-grained Hamiltonian model in the more realistic setting of having access only to the slow variables, as the perturbation level $\varepsilon$, the noise level $\sigma _{NR}$, and the region of phase space are varied.

## Numerical experiments

To exhibit the efficacy of weak-form coarse-graining in a variety of contexts, we examine the following nearly periodic Hamiltonian systems. In each case we apply the geometric reduction procedure outlined in Section “Hamiltonian structure of first-order averaging theory” to derive the reduced Hamiltonian system to be discovered. Note that in Examples 1 and 2 the coefficients of the reduced system are given in terms of special functions and integrals which must be numerically evaluated.

### Example 1: nonlinearly coupled oscillators

**Figure 3:**
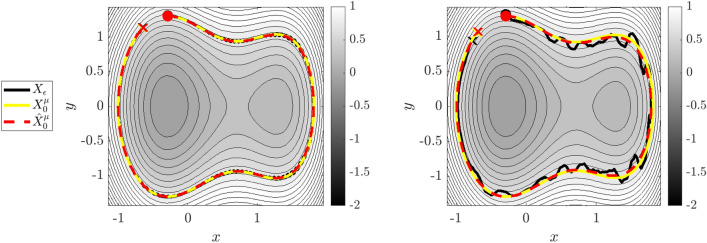
Reduced Dynamics of Example 1. True dynamics $X_\varepsilon$ are shown in black, leading-order reduced dynamics $X^{\mu }_0$ in yellow, and learned reduced dynamics $\widehat{X}^{\mu }_0$ in red (initial and final conditions are marked with dots and x’s), overlaying contours of the learned reduced Hamiltonian $\widehat{{\mathcal {H}}}^\mu _0$. Left: $\varepsilon = 0.01$ dynamics, right: $\varepsilon =0.05$. At $\varepsilon =0.05$ the nonlinear coupling between the fast and slow oscillators results in irregular perturbations to the slow variables.

For our first system we examine two coupled oscillators with different timescales and a nonlinear coupling potential. The $\varepsilon$-dependent Hamiltonian and sympletic form on $\mathbb {R}^4$ are given by

28 $$\begin{aligned} H_\varepsilon (Q,P,q,p) = \frac{1}{2} (Q^2 +P^2 ) + \frac{1}{2} \varepsilon (q^2 + p^2 ) + \varepsilon V(Q,q), \quad \Omega = dQ\wedge dP+dq\wedge dp \end{aligned}$$

with coupling potential given by

$$\begin{aligned} V(Q,q) = Qq \sin {(2Q + 2q)}. \end{aligned}$$

Note that $\Omega _\varepsilon = \Omega _0=\Omega$. The variables (*Q*, *P*) denote the position and momentum of a fast oscillator with dynamics on ${\mathcal {O}}(1)$ timescales, while (*q*, *p*) denotes the slow variables evolving on a timescale of ${\mathcal {O}}(\varepsilon )$. The equations of motion for this two-oscillator system are

29 $$\begin{aligned} {\left\{ \begin{array}{ll} \dot{Q} = P, \qquad &{} \dot{P} = -Q - \varepsilon \partial _{Q} V(Q,q) = -Q - \varepsilon \,\Big (q\,\sin {(2Q + 2q)} + 2\,Q\,q\,\cos (2Q + 2q)\Big ) \\ \dot{q} = \varepsilon p, \qquad &{} \dot{p} = -\varepsilon q - \varepsilon \partial _{q} V(Q,q) = -\varepsilon \,q - \varepsilon \Big (Q\, \sin {(2Q + 2q)} + 2\,Q\,q\,\cos (2Q+2q)\Big ). \end{array}\right. } \end{aligned}$$

As $\varepsilon$ increases, the fast variables (*Q*, *P*) impart large perturbations to the oscillating motion of the slow variables (*q*, *p*) by means of *V* (see Fig. 3, right). At $\varepsilon = 0$ the two oscillators decouple and the dynamics are governed by the limiting roto-rate

$$\begin{aligned} R_0 = P\partial _Q-Q\partial _P, \end{aligned}$$

which we identify as the Hamiltonian flow on $(\mathbb {R}^4,\Omega )$ of the leading-order adiabatic invariant

$$\begin{aligned} \mu _0(Q,P,q,p) = \frac{1}{2}(Q^2 + P^2). \end{aligned}$$

In this case the limiting frequency function $\omega _0$ such that $X_0=\omega _0R_0$ is unity. Hence, to leading order (*Q*, *P*) exhibits periodic clockwise circular rotation while (*q*, *p*) remains fixed. The flow-map $\Phi _t^0$ for $R_0$ is thus given by

30 $$\begin{aligned} \begin{pmatrix} Q\\ P\end{pmatrix} \mapsto {\mathcal {R}}(t)\begin{pmatrix} Q\\ P\end{pmatrix}, \quad \begin{pmatrix} q\\ p\end{pmatrix} \mapsto \begin{pmatrix} q\\ p\end{pmatrix};\qquad \qquad {\mathcal {R}}(t) = \begin{pmatrix} \cos (t) &{} \sin (t) \\ -\sin (t) &{} \cos (t)\end{pmatrix}. \end{aligned}$$

Each level set of $\mu _0$ is a single orbit of $R_0$ crossed with the slow variables $(q,p)\in \mathbb {R}^2$, hence the quotient procedure $M\rightarrow \Lambda _\mu \rightarrow M^{\mu }_0$ produces $M^{\mu }_0=(q,p)\in \mathbb {R}^2$. Thus, the leading-order reduced Hamiltonian ${\mathcal {H}}^{\mu }_0(q,p)$, is obtained from averaging $H_\varepsilon$ around $\Phi _t^0$ starting from an arbitrary point (*Q*, *P*, *q*, *p*) satisfying $\mu = \mu _0(Q,P,q,p)$:

$$\begin{aligned} {\mathcal {H}}^{\mu }_0(q,p)&= \frac{1}{2\pi }\int _0^{2\pi } H_\varepsilon (\Phi _t^0(Q,P,q,p))\,dt,\\&=\mu + \varepsilon \, \frac{1}{2}(q^2 + p^2) + \varepsilon \,\frac{1}{2\pi } \int _0^{2\pi }{V(Q \cos {t} + P \sin {t} , q) dt} \\&= \mu + \varepsilon \, \frac{1}{2}(q^2 + p^2) + \varepsilon \,\frac{q}{2\pi }\int _0^{2\pi }{ \left( Q \cos {t} + P \sin {t} \right) \sin {\left( 2\left( Q \cos {t} + P \sin {t} \right) + 2q \right) } dt} \end{aligned}$$

which can has an analytical form in terms of the Bessel function of the first kind $\mathcal {J}_1(z)$,

31 $$\begin{aligned} {\mathcal {H}}^{\mu }_0(q,p)= \mu + \varepsilon \, \frac{1}{2}(q^2 + p^2) + \varepsilon \sqrt{ 2\mu } \ \mathcal {J}_1\left( 2 \sqrt{ 2\mu } \right) \,Q \cos (2 Q). \end{aligned}$$

The leading-order reduced equations of motion for the slow variables (*q*, *p*) are therefore given by

32 $$\begin{aligned} {\left\{ \begin{array}{ll} \dot{q} = \partial _{p}{\mathcal {H}}^{\mu }_0=\varepsilon \,p\\ \dot{p} = -\partial _{q}{\mathcal {H}}^{\mu }_0= -\varepsilon \,q - \varepsilon \,\sqrt{ 2\mu } \ \mathcal {J}_1\left( 2 \sqrt{ 2\mu } \right) \,\cos (2q) + 2\,\varepsilon \,\sqrt{ 2\mu } \ \mathcal {J}_1\left( 2 \sqrt{ 2\mu } \right) \,q\,\sin (2q). \end{array}\right. } \end{aligned}$$

### Example 2: Hénon–Heiles embedded pendulum

**Figure 4:**
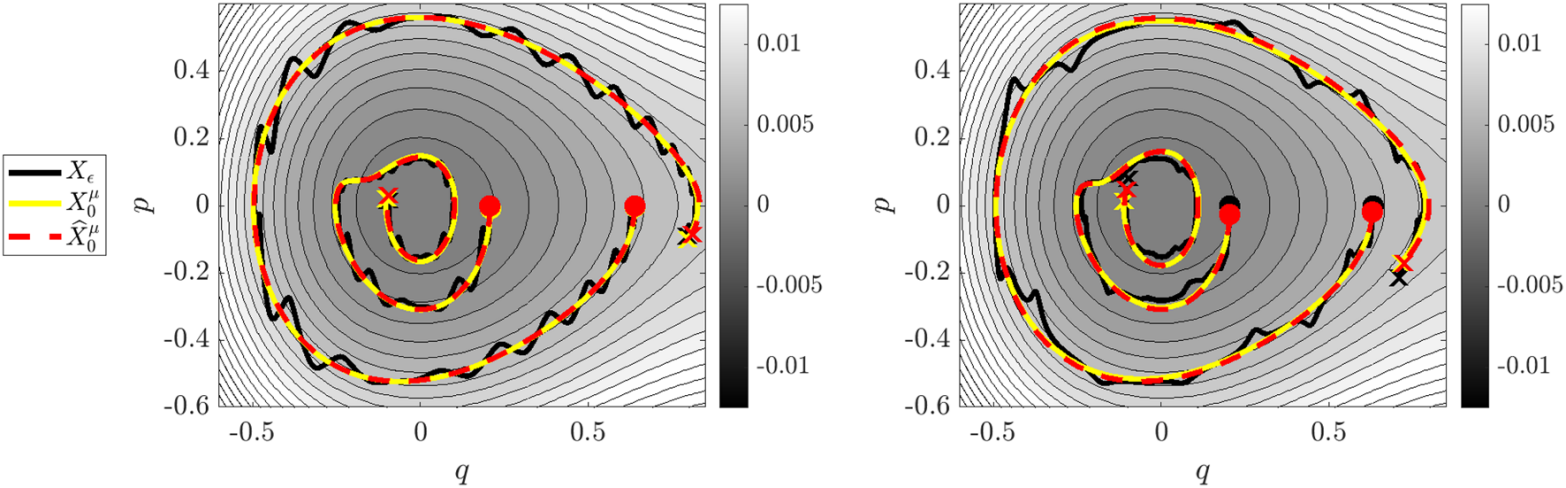
Reduced Dynamics of Example 2. Here $\varepsilon = 0.05$ and different initializations of the embedded pendulum are shown. Left: $(Q(0),P(0)) = (\frac{1}{2}\pi ,0)$, with pendulum dynamics close to linear. Right: $(Q(0),P(0)) = (\frac{31}{32}\pi ,0)$, with the pendulum initialized close to the unstable fixed, removing the strict scale separation (see Fig. 5). For each, $(q_1,p_1)$ and $(q_2,p_2)$ are plotted as separate trajectories overlaying $\widehat{{\mathcal {H}}}^\mu _0(0,0,q_2,p_2)$ (coloring conventions are the same as in Fig. 3).

Let $z = (Q,P,q_1,p_1,q_2,p_2)$ denote the variables of a 3-degree of freedom system with Hamiltonian

$$\begin{aligned} H_\varepsilon (z)&= H_0(z) + \varepsilon \,H_1(z)\\&= \frac{1}{2}P^2 + \alpha ^2\,(1-\cos \,Q) + \varepsilon \left( \frac{1}{2}p_1^2 + \frac{1}{2}\nu _1(Q)q_1^2 + \frac{1}{2}p_2^2 + \frac{1}{2}\nu _2(Q)\,q_2^2 + V(q_1,q_2,Q)\right) , \end{aligned}$$

and canonical symplectic form $\Omega = dQ\wedge dP + dq_1\wedge dp_1 + dq_2 \wedge dp_2$, again independent of $\varepsilon$. Here $\alpha ^2$ is a positive constant and $\nu _1:\mathbb {R}\rightarrow \mathbb {R}$, $\nu _2:\mathbb {R}\rightarrow \mathbb {R}$, $V:\mathbb {R}^3\rightarrow \mathbb {R}$ are each fixed smooth functions. The corresponding equations of motion are

33 $$\begin{aligned} {\left\{ \begin{array}{ll} \dot{Q} = P, \quad &{}\dot{P} = -\alpha ^2\,\sin \,Q - \varepsilon \,\bigg (\frac{1}{2}\frac{d\nu _1}{dQ}\,q_1^2 + \frac{1}{2}\frac{d\nu _2}{dQ}\,q_2^2 + \partial _QV\bigg )\\ \dot{q}_1 = \varepsilon \,p_1, \quad &{}\dot{p}_1 = - \varepsilon \Big (\nu _1(Q)\,q_1 +\partial _{q_1}V\Big )\\ \dot{q}_2 = \varepsilon \,p_2, \quad &{} \dot{p}_2 = -\varepsilon \Big (\nu _2(Q)\,q_2 + \partial _{q_2}V\Big ) \end{array}\right. } \end{aligned}$$

On the subset of phase space $S_0 = \left\{ z\ :\ \frac{1}{2}P^2 < \alpha ^2(1+\cos \,Q)\right\}$, (33) defines a fast-slow system. The limiting angular frequency function (see Definition 2.5) is

34 $$\begin{aligned} \omega _0(z) =\frac{4}{\alpha }\int _0^1\frac{ds}{\sqrt{1 - \frac{H_0(z)}{2\alpha ^2}s^2}\sqrt{1-s^2}}= \frac{4}{\alpha }K\left( {\frac{H_0(z)}{2\alpha ^2}}\right) \end{aligned}$$

where *K* denotes the complete elliptic integral of the first kind (defined in e.g.^41^; note that within $S_0$ the argument for *K* is in the interval [0, 1]). The limiting roto-rate is

$$\begin{aligned} R_0 = -\frac{\alpha ^2}{\omega _0}\,\sin Q\,\partial _{P} + \frac{P}{\omega _0}\,\partial _Q. \end{aligned}$$

Let $\Phi _\theta$ denote the time-$\theta$ flow map for $R_0$. The leading-order adiabatic invariant is given by

$$\begin{aligned} \mu _0(z)&= \bigg (\frac{1}{2\pi }\int _0^{2\pi }\Phi _\theta ^*(P\,dQ + p_1\,dq_2 + p_2\,dq_2)\,d\theta \bigg )_z(R_0(z))\\&= \frac{1}{2\pi }\int _0^{2\pi }(P\,dQ + p_1\,dq_2 + p_2\,dq_2)_{\Phi _\theta (z)}(T_z\Phi _\theta R_0(z))\,d\theta \\&= \frac{1}{2\pi }\int _0^{2\pi } \bigg (\iota _{R_0}(P\,dQ + p_1\,dq_2 + p_2\,dq_2)\bigg )(\Phi _\theta (z))\,d\theta \\&= \frac{1}{2\pi }\int _{\gamma (z)}P\,dQ. \end{aligned}$$

where $\gamma (z)$ is the parameterized curve $\gamma (z)(\theta ) = \Phi _\theta (z)$. This integral can also be expressed in terms of elliptic integrals as

$$\begin{aligned} \mu _0(z) = 16\,\alpha \,E\left( {\frac{H_0(z)}{2\alpha ^2}}\right) + \left( 8\frac{H_0(z)}{\alpha } - 16\,\alpha \right) K\left( {\frac{H_0(z)}{2\alpha ^2}}\right) , \end{aligned}$$

where *E* denotes the complete elliptic integral of the second kind.

Like Example 1, $\mu _0$ is only a function of the fast variables (*Q*, *P*). For *Q* mod 2$\pi$, level sets of $\mu _0$ contain a single $R_0$ orbit, so again the quotient procedure $M\rightarrow \Lambda _\mu \rightarrow M^{\mu }_0$ simply eliminates the fast variables (*Q*, *P*). Let $(Q_\theta ,P_\theta )$ be defined by $\Phi _\theta (z) = (Q_\theta ,q_1,q_2,P_\theta ,p_1,p_2)$. The first-order averaged Hamiltonian $H_\varepsilon ^*$ and reduced Hamiltonian ${\mathcal {H}}^{\mu }_0$ are then given by

$$\begin{aligned} H_\varepsilon ^*(z) = H_0(z)+\varepsilon \,\bigg (\frac{1}{2}p_1^2 + \frac{1}{2}\overline{\nu }_1(\mu )\,q_1^2 + \frac{1}{2}p_2^2 + \frac{1}{2}\overline{\nu }_2(\mu )\,q_2^2 +\overline{V}(q_1,q_2,\mu ) \bigg ) = H_0(z) +\varepsilon {\mathcal {H}}^{\mu }_0(q_1,p_1,q_2,p_2) \end{aligned}$$

where

35 $$\begin{aligned} \overline{\nu }_1(\mu ) = \frac{1}{2\pi }\int _0^{2\pi } \nu _1(Q_\theta )\,d\theta ,\quad \overline{\nu }_2(\mu ) = \frac{1}{2\pi }\int _0^{2\pi } \nu _2(Q_\theta )\,d\theta ,\quad \overline{V}(q_1,q_2,\mu ) = \frac{1}{2\pi }\int _0^{2\pi }V(q_1,q_2,Q_\theta )\,d\theta , \end{aligned}$$

with the integrals taken along any trajectory $(Q_\theta ,P_\theta )$ of $R_0$ with $\mu _0(Q_0,P_0) = \mu$.

We choose the following parametrizations, which lead to the reduced dynamics reproducing the famous Hénon–Heiles system^42^ proposed as a simple model for the motion of a star within a galaxy:

36 $$\begin{aligned} \nu _1(Q) = \nu _2(Q)=\nu _3(Q)=1 + 2\sin (Q),\quad V(Q,q_1,q_2) = \nu _3(Q)\left( q_1^2\,q_2 -\frac{1}{3} q_2^3\right) . \end{aligned}$$

**Figure 5:**
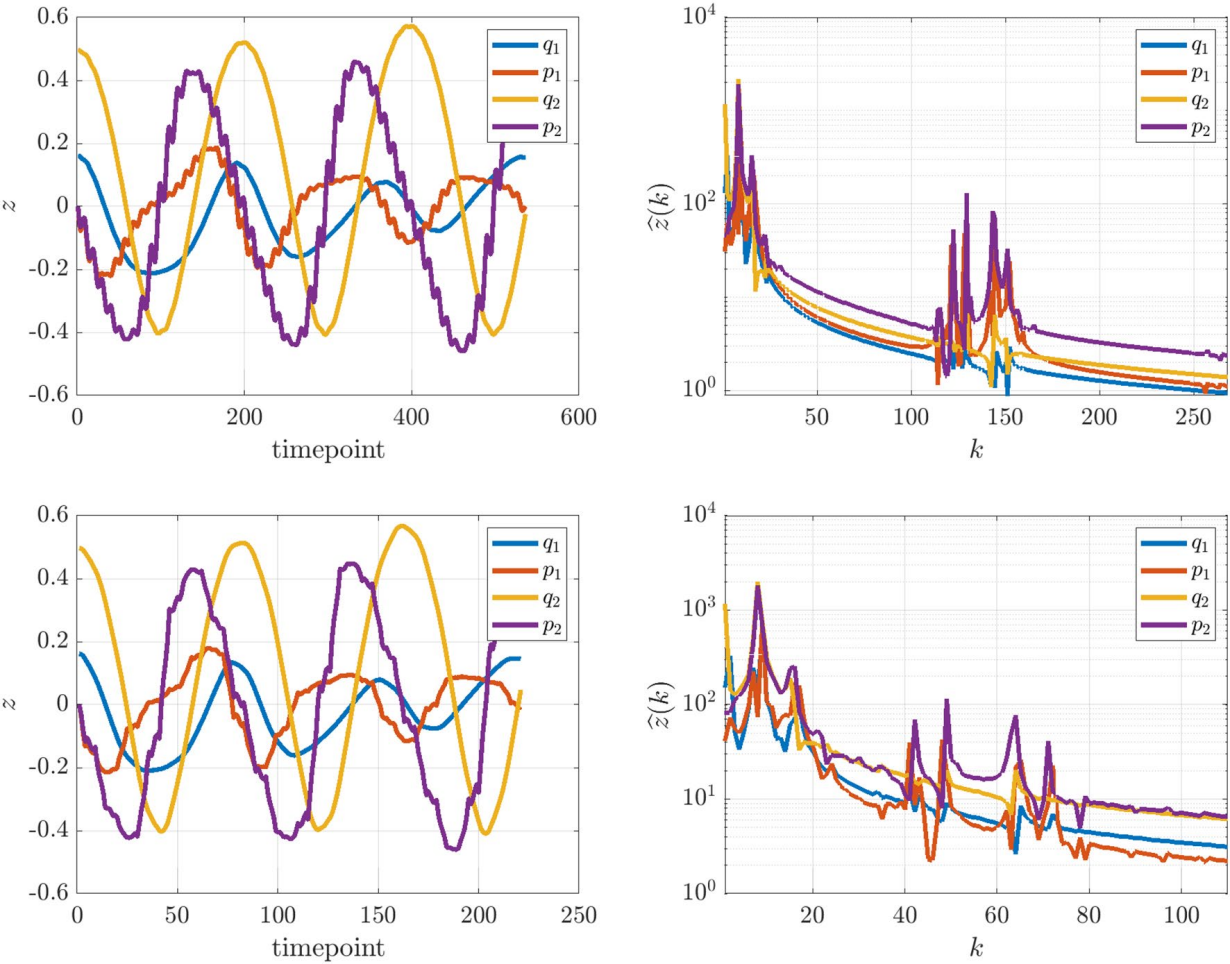
Lack of scale separation in Example 2. Time series of the slow variables for Example 2 with $\varepsilon =0.05$ (left) along with power spectra (right). The top plots show $Q(0) = \pi /2$, and an observable separation of scales (top right). The separation of scales breaks down at $Q(0) = 31\pi /32$ (bottom row) as the embedded pendulum dynamics approach the separatrix, as observed in the power spectrum.

The dynamics for two different initializations of the fast nonlinear pendulum are visible in Fig. 4. Unlike the previous example, here the nonlinear pendulum dynamics lead to anisotropic oscillation frequencies depending on the initial conditions. Correspondingly, the identified reduced system is highly-dependent on which integral curve of the roto-rate the initial conditions lie. For (*Q*(0), *P*(0)) close to the unstable fixed-point $Q(0) = \pi$, the fast dynamics become increasingly nonlinear and exhibit a longer period. This leads to a blending of the fast and slow scales, as visualized in Fig. 5 in Fourier space. The right plots show the power spectrum of the trajectories at $Q(0) = \pi /2$ (top) and $Q(0)=31\pi /32$ (bottom), where the latter clearly has much less of a separation of scales. This example demonstrates that WSINDy is still able to identify the leading-order reduced dynamics from systems with a complex microstructure.

### Example 3: Charged particle motion

**Figure 6:**
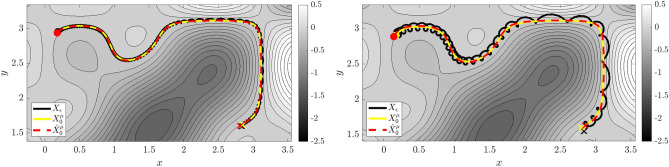
Reduced Dynamics of Example 3. Left: $\varepsilon = 0.01$ dynamics, right: $\varepsilon =0.03$. On the right one can observe irregular perturbations as the unreduced dynamics (black curve) jump between contours of the electric potential energy $\varphi$ shown in gray. (Coloring conventions are the same as in Fig. 3).

Consider the motion of a charged particle with position (*x*, *y*, *z*) and velocity $(v_x,v_y,v_z)$ in a time-independent electromagnetic field of the form $\varvec{B}(x,y,z) = \nabla \times \textbf{A}(x,y,z)$, $\varvec{E}(x,y,z) = -\nabla \varphi (x,y,z)$. If the field potentials are independent of *z*, that is, $\textbf{A}= (A_x(x,y),A_y(x,y),0)$ and $\varphi = \varphi (x,y)$, then the *z*-component of the particle momentum is a constant of motion. Therefore the Lorentz force equation describing the particle’s motion may be written, for $B(x,y) = \partial _xA_y-\partial _yA_x$, as

37 $$\begin{aligned} {\left\{ \begin{array}{ll} \dot{x} = \varepsilon \,v_x\,, \quad &{} \dot{v}_x = -\partial _x\varphi (x,y) +v_y\,B(x,y)\\ \dot{y} = \varepsilon \,v_y\,,\quad &{} \dot{v}_y = -\partial _y\varphi (x,y) - v_x\,B(x,y) \end{array}\right. } \end{aligned}$$

This is an $\varepsilon$-dependent Hamiltonian system on $\mathbb {R}^4\ni (x,y,v_x,v_y)$ if the Hamiltonian and symplectic form are given by

$$\begin{aligned}&H_\varepsilon (x,y,v_x,v_y) = \varepsilon ^2\,\frac{1}{2}(v_x^2 + v_y^2 ) + \varepsilon \,\varphi (x,y) \\&\Omega _\varepsilon = -B(x,y)\,dx\wedge dy + \varepsilon \,(dx\wedge dv_x + dy\wedge dv_y). \end{aligned}$$

Letting $q = (x,y)$, $v = (v_x,v_y)$, the flow map of the roto-rate at $\varepsilon =0$ is given by

38 $$\begin{aligned} \Phi _t^0(q,v) =\Big ( q,\ D(q) + {\mathcal {R}}(t)(v-D(q))\Big ), \qquad D(q):= (-\partial _y\varphi / B,\ \partial _x\varphi / B) \end{aligned}$$

with ${\mathcal {R}}$ the rotation matrix from Example 1. Using the formulas in^16^, the adiabatic invariant is degenerate to first order, with $\mu _* = \mu _2$ (see (14)) given by

$$\begin{aligned} \mu _2(q,v)&= \frac{1}{2}\frac{|v - D(q)|^2}{B(q)}. \end{aligned}$$

The first-order averaged system $X_\varepsilon ^*$ is automatically presymplectic Hamiltonian according to Lemma 2, with Hamilton’s equations (15) for $H_\varepsilon ^*=\varepsilon \,\varphi (x,y)$ and $\Omega _0=- B(x,y)\,dx\wedge dy$ producing the first-order averaged dynamics

39 $$\begin{aligned} \dot{x} = -\varepsilon \,\frac{\partial _y \varphi (x,y)}{B(x,y)}, \qquad \dot{y} = \varepsilon \,\frac{\partial _x \varphi (x,y)}{B(x,y)}. \end{aligned}$$

Restriction to a level set of $\mu _2$ merely restricts *v*-space, which is already absent from dynamics, and the flow of the roto-rate fixes *q*-variables, so we have ${\mathcal {H}}^{\mu }_0= H_\varepsilon ^*$, $\Omega ^{\mu }_0= \Omega _\varepsilon ^*$.

A simple test case for this problem is a constant magnetic field. This corresponds physically to zero current density, as this reduces Ampére’s law to $\nabla \times \pmb {B} = 0$, under which the assumption $\pmb {B} = B(x,y)\hat{z}$ leads to $B = const$. We set $B=1$ throughout, which for example corresponds to the vector potential $\textbf{A}=(\frac{1}{2}x,-\frac{1}{2}y,0)$. We prescribe a sinusoidal ambient electric field potential

40 $$\begin{aligned} \varphi (x,y) = \sum _{j=1}^3 \frac{1}{j} \sin (jx)\sin (jy) \end{aligned}$$

which, given Gauss’s law $\Delta \varphi = \varepsilon _0^{-1}\rho$ relating the electric potential $\varphi$ to the charge density $\rho$, loosely corresponds to a lattice of bound charges.

As the charged particle approximately travels along contours of $\phi$, the velocity variables $(v_x,v_y)$ exhibit near-periodic fast-scale oscillations. For larger $\varepsilon$, the motion of $(v_x,v_y)$ becomes modulated by the steepness of $\nabla \phi$, which leads to irregular perturbations to the position variables (*x*, *y*). This can be seen in Fig. 6 (right) as the oscillations in the black curve become elongated.

### Example 4: Coupled charged particle motion

**Figure 7:**
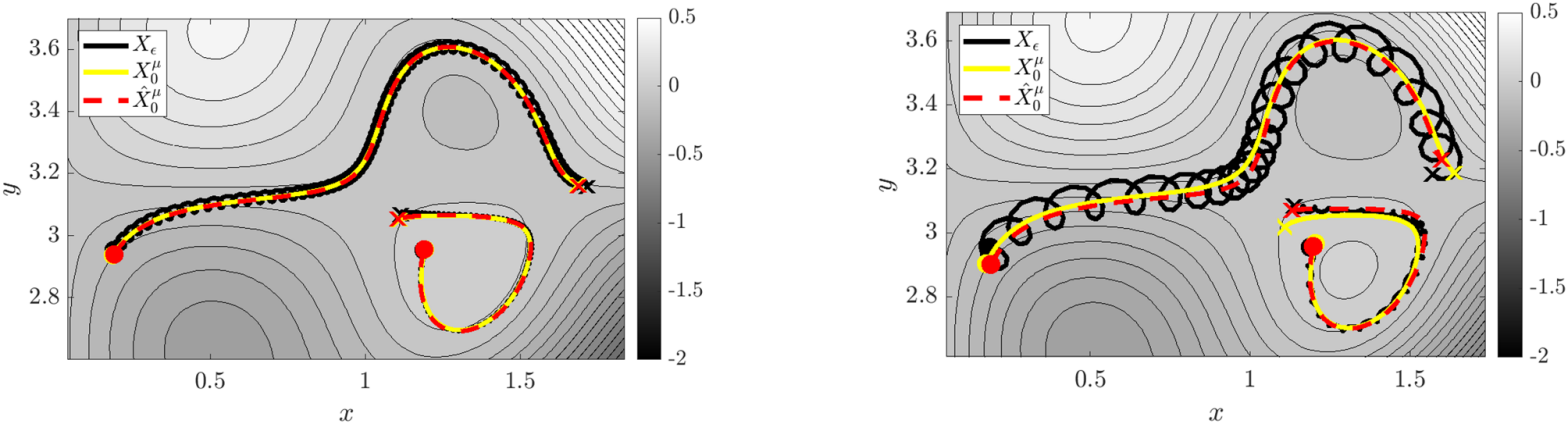
Reduced Dynamics of Example 4. Left: $\varepsilon = 0.01$ dynamics, right: $\varepsilon =0.03$, overlaying contours of the learned background electric field. Particle two, initialized at $(x_2,y_2) = (1.2,3.0)$, has zero initial velocity and hence smaller perturbations as a result of the Lorentz force.

The previous example outlines a method of reducing the dimensionality of certain systems by a factor of two. We extend it in our last example to a system of two particles $\{(x_1,y_1,v_{x_1},v_{y_1})$, $(x_2,y_2,v_{x_2},v_{y_2})\}\in \mathbb {R}^{4\times 4}\cong \mathbb {R}^8$ interacting under a pair-wise potential *K* as well as the same background fields $(\pmb {E},\pmb {B})$ in the previous example. The dynamics are illustrated in Fig. 7 for $\varepsilon =0.01$ and $\varepsilon =0.03$. The Hamiltonian describing this motion is

$$\begin{aligned} H_\varepsilon&= \varepsilon ^2\,\frac{1}{2}(v_{x_1}^2 + v_{y_1}^2 +v_{x_2}^2 + v_{y_2}^2 ) + \varepsilon \,(\varphi (x_1,y_1) + \varphi (x_2,y_2) + K(x_1,y_1,x_2,y_2)). \end{aligned}$$

with symplectic form

$$\begin{aligned} \Omega _\varepsilon&= -\,dx_1\wedge dy_1 -\,dx_2\wedge dy_2 + \varepsilon \,(dx_1\wedge dv_{x_1} + dy_1\wedge dv_{y_1}+dx_2\wedge dv_{x_2} + dy_2\wedge dv_{y_2}). \end{aligned}$$

If *K* is the Coulomb potential in 2*D*, $K_c(x_1,y_1,x_2,y_2) = Q\log \left( \sqrt{(x_1-x_2)^2+(y_1-y_2)^2}\right)$, the system may be used to classically describe a pair of charged particles (*Q* corresponding to the product of two charges). To regularize the system we use a crude approximation to $K_c$ given by the first-order Taylor expansion about unit separation

$$\begin{aligned} K(x_1,y_1,x_2,y_2)= -\frac{Q}{2}\big (1 - (x_1-x_2)^2-(y_1-y_2)^2\big ), \end{aligned}$$

which provides a reasonable approximation for interparticle distances near unity. We use a repulsive version of this interaction potential with $Q = -(2\pi )^{-1}$, under which the dynamics become

41 $$\begin{aligned} {\left\{ \begin{array}{ll} \dot{x_1} = \varepsilon \,v_{x_1},\quad &{} \dot{v}_{x_1} = -\partial _{x_1}\varphi (x_1,y_1)+\frac{1}{2\pi }(x_1-x_2) +v_{y_1}\\ \dot{y_1} = \varepsilon \,v_{y_1}, \quad &{} \dot{v}_{y_1} = -\partial _{y_1}\varphi (x_1,y_1)+\frac{1}{2\pi }(y_1-y_2) - v_{x_1}\\ \dot{x_2} = \varepsilon \,v_{x_2}, \quad &{} \dot{v}_{x_2} = -\partial _{x_2}\varphi (x_2,y_2)+\frac{1}{2\pi }(x_2-x_1) +v_{y_2}\\ \dot{y_2} = \varepsilon \,v_{y_2}, \quad &{}\dot{v}_{y_2} = -\partial _{y_2}\varphi (x_2,y_2)+\frac{1}{2\pi }(y_2-y_1) - v_{x_2} \end{array}\right. } \end{aligned}$$

where $\varphi$ is given by (40). This leads to the leading order roto-rate:

$$\begin{aligned} R_0 = (-\partial _{x_1}\varphi (x_1,y_1)+\frac{1}{2\pi }(x_1-x_2) +v_{y_1})\partial _{v_{x_1}} +(-\partial _{y_1}\varphi (x_1,y_1)+\frac{1}{2\pi }(y_1-y_2) - v_{x_1})\partial _{v_{y_1}} \\ + \ (-\partial _{x_2}\varphi (x_2,y_2)+\frac{1}{2\pi }(x_2-x_1) +v_{y_2})\partial _{v_{x_2}} +(-\partial _{y_2}\varphi (x_2,y_2)+\frac{1}{2\pi }(y_2-y_1) - v_{x_2})\partial _{v_{y_2}}. \end{aligned}$$

Carrying out the same procedures as before (note that presymplectic Hamiltonian dynamics also appear), we arrive at the reduced Hamiltonian

$$\begin{aligned} {\mathcal {H}}^{\mu }_0(x_1,y_1,x_2,y_2) = \varepsilon \left( \varphi (x_1,y_1)+\varphi (x_2,y_2) +K(x_1,y_1,x_2,y_2)\right) \end{aligned}$$

which under the reduced symplectic form $\Omega ^{\mu }_0= -dx_1\wedge dy_1-dx_2\wedge dy_2$ gives us the reduced Hamiltonian dynamics in $\mathbb {R}^4$,

42 $$\begin{aligned} {\left\{ \begin{array}{ll} \dot{x_1} = \varepsilon \,\left( -\partial _{y_1}\varphi (x_1,y_1)+\frac{1}{2\pi }(y_1-y_2)\right) , \quad &{} \dot{y_1} = \varepsilon \,\left( \partial _{x_1}\varphi (x_1,y_1)-\frac{1}{2\pi }(x_1-x_2)\right) ,\\ \dot{x_2} = \varepsilon \,\left( -\partial _{y_2}\varphi (x_2,y_2)+\frac{1}{2\pi }(y_2-y_1) \right) , \quad &{} \dot{y_2} = \varepsilon \,\left( \partial _{x_2}\varphi (x_2,y_2)-\frac{1}{2\pi }(x_2-x_1)\right) . \end{array}\right. } \end{aligned}$$

### Dynamics and sampling regimes

Since we are interested in identifying coarse-grained models, we focus on observing only the slow variables and attempt to recover ${\mathcal {H}}^{\mu }_0$. To exemplify a range of conditions under which WSINDy identifies the correct coarse-grained dynamics, we quantify the accuracy between the learned ($\widehat{{\mathcal {H}}}^\mu _0$) and true (${\mathcal {H}}^{\mu }_0$) reduced Hamiltonian under the following settings:

(i) **Regions of phase space:** We sample trajectories with initial conditions approaching an elliptic fixed point of ${\mathcal {H}}^{\mu }_0$, near which the $\varepsilon =0$ dynamics dominate and ${\mathcal {H}}^{\mu }_0$ fails to be recovered, to probe the near-periodicity of the system. (For Example 4, one particle is placed near an elliptic fixed point and the other a distance one away with zero velocity).
(ii) **Perturbative regime:** We examine two perturbative regimes, labeled the *mild* and *extreme* regimes. For Examples 1,3, and 4 this is defined by two different values of $\varepsilon$, with $\varepsilon \in \{0.01,0.05\}$ for Example 1 and $\varepsilon \in \{0.01,0.03\}$ for Examples 3 and 4. For Example 2 we fix $\varepsilon$ at the reasonably large value $\varepsilon =0.05$ and define the mild and extreme regimes as the two different sets of initial conditions for the nonlinear pendulum, $(Q(0),P(0)) \in \{(\frac{\pi }{2},0),(\frac{31\pi }{32},0)\}$, the latter driving the system very close to the separatrix at $Q=\pi$, whereby timescale separation disappears (see Fig. 5).
(iii) **Data sampling regime:** We fix the timestep $\Delta t$ to be 10 points per fast cycle and the total time window *T* to be 4 cycles of the slow system, or with $T_s,T_f$ denoting dominant slow and fast periods,
  $$\begin{aligned} T = 4T_s, \qquad \Delta t = T_f / 10. \end{aligned}$$
  From a practical perspective, this is realistic because one cannot expect to sample the fast system at a high resolution; however, this level of resolution is still adversarial because the sampling rate is high enough for WSINDy to resolve the fast system (as evidenced by Figs. 1, 2). With coarser sampling one might expect to more easily recover the reduced dynamics via aliasing, a property leveraged in^33^, although in general, larger $\varepsilon$ leads to mixing of the slow and fast scales over longer time windows. This is why the test function support in Example 2 cannot be taken too large (see Figure S2, row 2, in the Supplemental Information), hence we conjecture that in general the aliasing approach will lead to inaccurate reduced dynamics. A total time window of 4 cycles ($T= 4T_s$) is used to provide enough data to identify a dominate slow scale (i.e. by sampling for longer than a single slow cycle). We also found that $T= 4T_s$ was necessary to identify the dynamics in Example 2 at $Q(0) = 31\pi /32$, as in this case the slow-fast period ratio is $T_{sf}:= T_s/T_f\approx 7$, leading to only 278 timepoints at the resolution $\Delta t = T_f/10$, $T = 4T_s$. For time windows $T\le 3T_s$ there simply is insufficient data for robust recovery in the high perturbative regime.
(iv) **Extrinsic noise:** Realistic measurement settings require consideration of extrinsic (measurement) noise. We define the noise level of the data by
  43 $$\begin{aligned} \sigma _{NR} = \frac{\left\| {\textbf{Z}^\star -\textbf{Z}} \right\| _2}{\left\| {\textbf{Z}^\star } \right\| _2} \end{aligned}$$
  where $\textbf{Z}^\star$ represents the exact (noise-free) data and $\textbf{Z}$ the observed (possibly noisy) data. We consider additive mean-zero i.i.d. Gaussian noise and noise levels up to $\sigma _{NR} = 0.1= 10\%$ noise, which together with the intrinsic perturbations imparted by the fast scale represents a significant level of corruption.

### Hyperparameters

The WSINDy hyperparameters consist mainly of the library $\mathbb {H}$ and test functions $\mathbb {V}$. The main purpose of this article is not to show robustness to sheer library size, but instead to show that under the weak-form transformations

$$\begin{aligned} \textbf{Z}\rightarrow \left\langle \dot{V},\textbf{Z}\right\rangle _\textbf{t}, \quad X_{H}(\textbf{Z}) \rightarrow \left\langle V, X_{H}(\textbf{Z})\right\rangle _\textbf{t}, \quad V\in \mathbb {V}, H\in \mathbb {H}\end{aligned}$$

the dynamics agree well with ${\mathcal {H}}^{\mu }_0$. For this reason, we restrict the library $\mathbb {H}$ to $40-70$ possible terms which include a representation of ${\mathcal {H}}^{\mu }_0$. In Examples 1-4, the leading-order reduced Hamiltonian ${\mathcal {H}}^{\mu }_0$ can be represented with trigonometric functions, polynomials and products thereof. Define the monomial library of degree *n* in 2*N* variables by

44 $$\begin{aligned} P^{(n)}_{2N} = \left\{ z\rightarrow \prod _{i=1}^{2N}z_i^{j_i}\,\ 1\le \sum _{i=1}^{2N} j_i\le n, j_i\in \mathbb {N}\cup \{0\}\right\} , \end{aligned}$$

and the partial cosine library with base frequency $f_0$ up to maximum frequency $nf_0$ as (discarding redundancies)

45 $$\begin{aligned} C^{(n,f_0)}_{2N}= \left\{ (q,p)\rightarrow \cos (jq_i+kp_i)\pm \cos (jq_i-kp_i)\,\ (j,k)\in \{0,f_0,\dots ,nf_0\}^2, i\in \{1,\dots ,N\}\right\} . \end{aligned}$$

Also define the product linear-trig library with trigonometric terms of frequencies $f_0$ to $nf_0$ by

46 $$\begin{aligned} LT^{(n,f_0)}_{2N} = \{z\rightarrow z_ig(kz_j)\,\ (i,j)\in \{1,\dots ,2N\}^2, g\in \{\cos ,\sin \}, k\in \{0,f_0,\dots ,nf_0\}\}. \end{aligned}$$

The libraries used in each example are combinations of $P^{(n)}_{2N}$, $C^{(n,f_0)}_{2N}$, and $LT^{(n,f_0)}_{2N}$, given in Table 1.

**Table 1:** Algorithmic specifications for examples. $\mathbb {H}$ denotes the Hamiltonian library employed in sparse regression (with $P^{(n)}_{2N}, C^{(n,f_0)}_{2N},LT^{(n,f_0)}_{2N}$ defined in (44)–(46)), $\#\{\mathbb {H}\}$ denotes the total number of terms in $\mathbb {H}$, ${\mathcal {H}}^{\mu }_0$ sparsity is the number of terms required to represent ${\mathcal {H}}^{\mu }_0$ from $\mathbb {H}$, and *Q* is the qauntity of interest used to measure agreement with ${\mathcal {H}}^{\mu }_0$ (see Eq. 48).

Example	$\mathbb {H}$	$\#\{\mathbb {H}\}$	${\mathcal {H}}^{\mu }_0$ sparsity	*Q*
1. Coupled Oscillators	$P^{(2)}_2\cup C^{(3,2)}_{2}\cup LT^{(3,2)}_2$	41	3	${\mathcal {H}}^{\mu }_0$
2. Hénon–Heiles Pendulum	$P^{(4)}_4$	69	6	${\mathcal {H}}^{\mu }_0(q_1,q_2,0,0)$
3. Charged particle	$C^{(4,1)}_{2}$	40	3	${\mathcal {H}}^{\mu }_0$
4. Coupled charged particles	$P^{(2)}_4\cup C^{(3,1)}_{4}$	62	12	${\mathcal {H}}^{\mu }_0(x_1,y_1,x_1,y_1)$

For the set of test vector fields $\mathbb {V}$, we take the simple convolutional approach as in^22^. That is, we fix a reference test function

$$\begin{aligned} \phi (t) = \exp \left( \frac{9}{(2t/T_\phi )^2-1}\right) \mathbbm {1}_{(-T_\phi /2,T_\phi /2)}(t), \end{aligned}$$

which is $C^\infty _c(\mathbb {R})$ and supported on $[-T_\phi /2,T_\phi /2]$ ($\mathbbm {1}_{S}$ denotes the indicator function on the set *S*). We then set the test vector fields to

$$\begin{aligned} V_k(z,t) = \phi (t-t_k)\sum _{j=1}^{2N} \partial _{z_j}, \qquad 1\le k\le K \end{aligned}$$

for a fixed set of *query timepoints* ${\mathcal {Q}}:= \{t_k\}_{k=1}^K$. The free parameters are ${\mathcal {Q}}$ and the support width $T_\phi$. It is clear from Fig. 2 that recovery of ${\mathcal {H}}^{\mu }_0$ depends on $\sigma _{\phi f}$ being sufficiently large. In Figure S2 of the Supplemental Information we demonstrate that this is ensured by choosing $T_\phi$ according to the Robust Corner Point method described in Section S1 of the Supplemental Information, a modification of the procedure in^22^. To summarize, the Robust Corner Point method in S1 is used to find a cornerpoint $k^*$ in the Fourier spectrum of the data, which is then assigned to lie 2 standard deviations into the tail of the power spectrum $|{\mathcal {F}}[\phi (\textbf{t})]|$, interpreted as a probability distribution over Fourier modes. This fixes the support width $T_\phi$. Here ${\mathcal {F}}$ is the discrete Fourier transform. We let ${\mathcal {Q}}$ be equally spaced and covering $\textbf{t}$ such that $T_\phi /(t_{k+1}-t_k) = 12$ for $1\le k\le K$.

### Performance metrics

We measure accuracy of the recovered Hamiltonian $\widehat{{\mathcal {H}}}^\mu _0$ with respect to ${\mathcal {H}}^{\mu }_0$ using three metrics, the *model selection accuracy*, *pointwise agreement with *${\mathcal {H}}^{\mu }_0$, and *forward simulation accuracy*. While all should be considered together to assess performance, each is independently useful and may suffice for specific applications. In what follows, let $\textbf{Z}$ denote the observed slow variables from $H_\varepsilon$, $\textbf{Z}^\mu$ the simulated data from ${\mathcal {H}}^{\mu }_0$, and $\widehat{\textbf{Z}}^\mu$ the simulated data from $\widehat{{\mathcal {H}}}^\mu _0$.

(i) *Model selection accuracy* is measured according to the *true positivity ratio* $\text {TPR}({\widehat{\textbf{w}}})$, defined as
  47 $$\begin{aligned} \text {TPR}({\widehat{\textbf{w}}}) = \frac{TP({\widehat{\textbf{w}}})}{TP({\widehat{\textbf{w}}})+FP({\widehat{\textbf{w}}})+FN({\widehat{\textbf{w}}})} \end{aligned}$$
  where *TP*, *FP*, and *FN* are the numbers of true nonzero entries, false nonzero entries, and false zero entries in ${\widehat{\textbf{w}}}$, respectively, compared to the correct weights $\textbf{w}^\star$ of ${\mathcal {H}}^{\mu }_0$. In this way, recovering *S* terms correctly and no false terms leads to $\text {TPR} = S/S^\star$ where $S^\star = \left\| {\textbf{w}^\star } \right\| _0$ is the sparsity of ${\mathcal {H}}^{\mu }_0$.
(ii) *Pointwise agreement with *${\mathcal {H}}^{\mu }_0$ is assessed using the relative error of a quantity of interest *Q*(*z*) related to each example,
  48 $$\begin{aligned} \Delta H({\widehat{\textbf{w}}}) = \left\| {Q -\widehat{Q}} \right\| _{\ell _2(D(\textbf{Z}))}/\left\| {Q} \right\| _{\ell _2(D(\textbf{Z}))} \end{aligned}$$
  where $\widehat{Q}$ is the learned quantity of interest, $D(\textbf{Z})$ is an equally-spaced computational grid covering the smallest rectangle in phase space containing the observed data $\textbf{Z}$, and $\left\| {Q} \right\| _{\ell _2(D(\textbf{Z}))} = \sqrt{\sum _{z\in D(\textbf{Z})} Q(z)^2}$. The quantities *Q* used for each example are listed in Table 1. For Examples 1 and 3 we simply take $Q = {\mathcal {H}}^{\mu }_0$. For Example 2 we use the zero-momentum section, $Q(z) = {\mathcal {H}}^{\mu }_0(q_1,q_2,0,0)$, which captures agreement with the potential field $\overline{V}$ defined in (36) and (35). In Example 4 we use agreement with the $(x_1,y_1) = (x_2,y_2)$ section, $Q(z) ={\mathcal {H}}^{\mu }_0(x_1,y_1,x_1,y_1)$, which measures agreement with the background electric field $\varphi (x,y)$.
(iii) *Forward simulation accuracy* of $\widehat{{\mathcal {H}}}^\mu _0$ is measured with respect to ${\mathcal {H}}^{\mu }_0$ and $H_\varepsilon$ using the following criteria.:
  49 $$\begin{aligned} \Delta \textbf{Z}^\mu&= \left\| {\textsf {vec}(\textbf{Z}^\mu - \widehat{\textbf{Z}}^\mu )} \right\| _2/\left\| {\textsf {vec}(\textbf{Z}^\mu )} \right\| _2, \end{aligned}$$
  50 $$\begin{aligned} \Delta \textbf{Z}&= \left\| {\textsf {vec}(\textbf{Z}- \widehat{\textbf{Z}}^\mu )} \right\| _2/\left\| {\textsf {vec}(\textbf{Z})} \right\| _2,\end{aligned}$$
  51 $$\begin{aligned} \Delta \textbf{Z}^\star&= \left\| {\textsf {vec}(\textbf{Z}- \textbf{Z}^\mu )} \right\| _2/\left\| {\textsf {vec}(\textbf{Z})} \right\| _2. \end{aligned}$$
  $\Delta \textbf{Z}^\mu$ is the primary forward simulation accuracy criteria, as it assesses agreement between the analytically-reduced and learned systems. $\Delta \textbf{Z}$ is not expected to be small for larger $\varepsilon$, but assesses whether the trajectory exhibits a phase error from the full system. $\Delta \textbf{Z}^\star$ is a reference measure between ${\mathcal {H}}^{\mu }_0$ and $H_\varepsilon$ (i.e. does not depend on the learning process), and serves as an approximate lower bound for $\Delta \textbf{Z}$.

Note that forward simulation accuracy is a more subtle performance metric because initial conditions for the reduced systems (${\mathcal {H}}^{\mu }_0$ and $\widehat{{\mathcal {H}}}^\mu _0$) are not simply the restriction of the initial conditions of $H_\varepsilon$ to the slow variables. In addition, the full dynamics may be chaotic, which may also lead to chaos in the reduced system if the reduced system has two or more degrees of freedom (e.g. Examples 2 and 4). To find accurate initial conditions we perform a minimal grid as described in the Supplemental Information, Section S3. To account for chaos, we only measure agreement up to the first full period of the observed variables, $T_s$, defined as the period of the dominant Fourier mode in the dynamics.

### Results: zero extrinsic noise ($\sigma _{NR}=0$)

**Figure 8:**
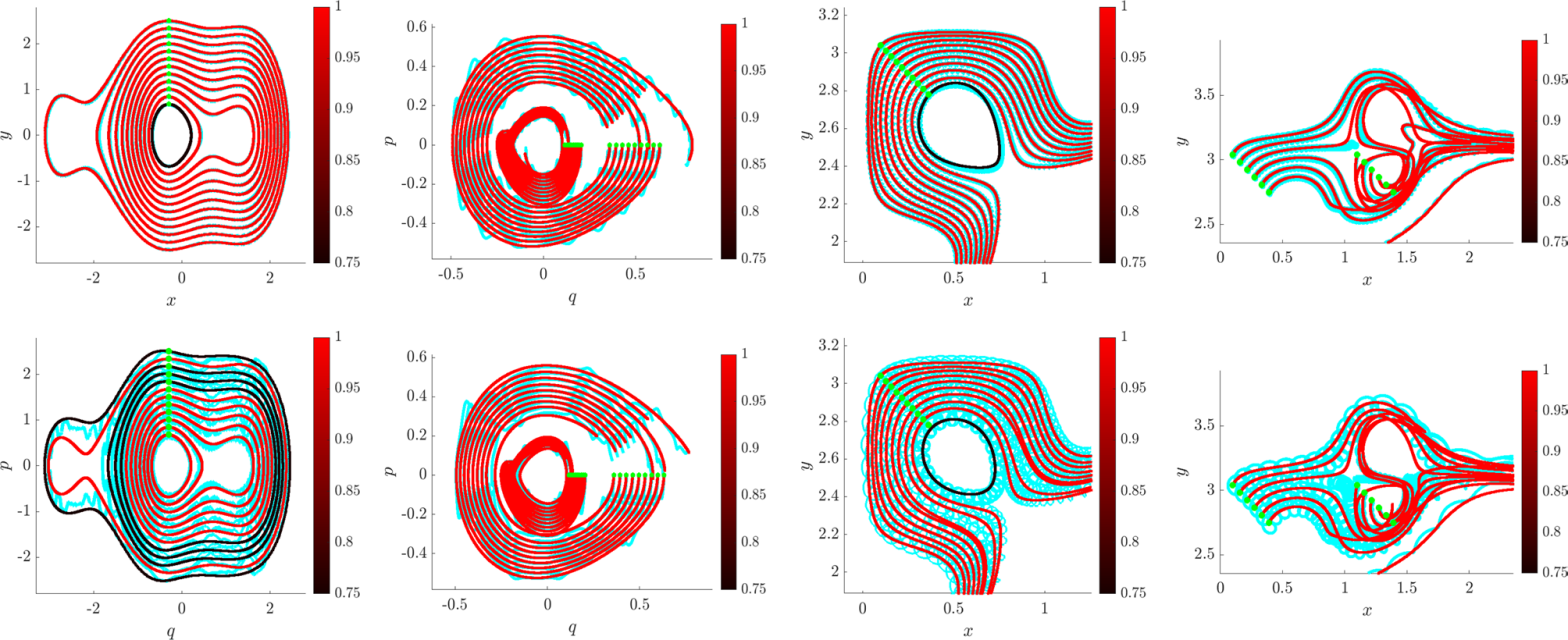
Model Selection Accuracy. TPR values (47) for Examples 1-4 (left to right) in the mild and perturbative regimes (top and bottom). Each trajectory $\widehat{\textbf{Z}}^\mu$ is simulated from its respective learned Hamiltonian system $\widehat{{\mathcal {H}}}^\mu _0$ and plotted on a red scale according to its TPR, overlaying the training data $\textbf{Z}$ in cyan. Green dots indicate initial conditions. The correct model is recovered in the vast majority of cases ($\text {TPR}=1$ in $91\%$ of trials). Exceptions in Examples 1 and 3 still produce accurate learned dynamics (for comparison with trajectories of the analytically reduced Hamiltonian ${\mathcal {H}}^{\mu }_0$, see Figures S5-S8 in the Supplemental Information).

**Figure 9:**
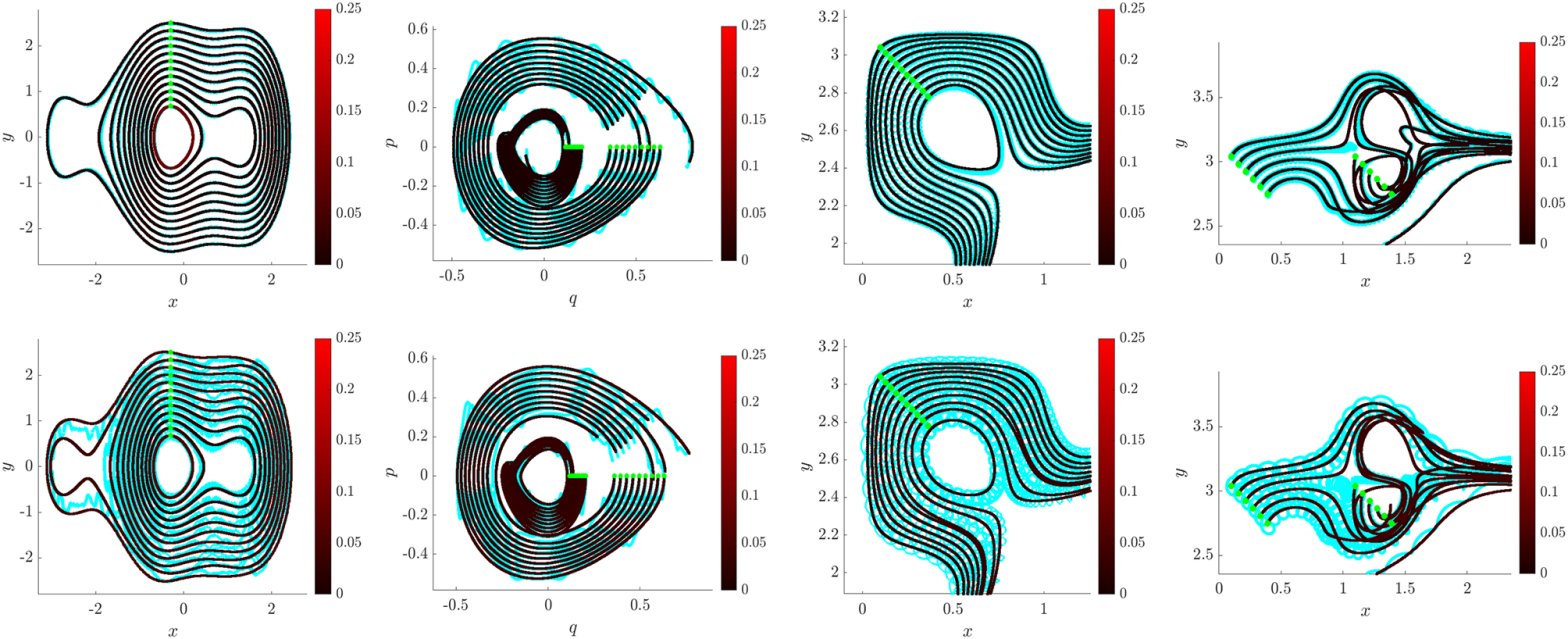
Pointwise agreement with ${\mathcal {H}}^{\mu }_0$. Same plot description as Fig. 8, now with color gradation according to $\Delta H$ (48). The learned Hamiltonian $\widehat{{\mathcal {H}}}^\mu _0$ agrees accurately with ${\mathcal {H}}^{\mu }_0$ in all trials ($\Delta H\le 0.053$ in all except one trial, see Table 2), including those with inexact model identification (see Fig. 8).

We now demonstrate that the reduced Hamiltonian ${\mathcal {H}}^{\mu }_0$ is sufficiently recovered over a wide range of initial conditions and perturbative regimes using a single trajectory from $H_\varepsilon$. This implies that only a small sample in phase space is needed to recover the entire reduced Hamiltonian, as opposed to neural-network based approaches, which are often trained on ${\mathcal {O}}(10^4)$ input-output pairs which cover phase space and require substantially longer computation times. The model selection accuracy (TPR, (47)) and pointwise agreement with ${\mathcal {H}}^{\mu }_0$ ($\Delta H$, (48)) are shown in Figs. 8 and 9, respectively, for Examples 1-4 (left to right) in the mild and extreme perturbative regimes (top and bottom rows). In each Figure, the learned trajectories $\widehat{\textbf{Z}}^\mu$ are plotted on a black-to-red scale overlaying the training data $\textbf{Z}$ in cyan. The color of each learned trajectory indicates the value of the given statistic (TPR or $\Delta H$). Table 2 contains numerical values for $\Delta H$ complementing Fig. 9. To minimize space, we have displayed forward simulation accuracy numerically in Table 3, and included corresponding Figures S5-S8 in the Supplemental Information.

#### Model Selection Accuracy (Figure 8)

In Fig. 8 we report the TPR score (defined in (47)) for each trajectory, indicating that in the vast majority of cases WSINDy recovers the correct terms in ${\mathcal {H}}^{\mu }_0$ even under significant perturbations imparted by the fast scale. The mild perturbative regime (top row) yields TPR=1 (i.e. perfect model recovery) for all cases except the inner-most trajectories of Examples 1 and 3, which are highly degenerate, nearly-circular orbits. Moreover, trajectories from these misspecified models show qualitative agreement with the true dynamics (see corresponding $\Delta H$ values in Fig. 9). Most surprisingly, there exist trajectories in Example 1 that enclose only one of the relevant fixed points and still enable identification of ${\mathcal {H}}^{\mu }_0$. In the extreme perturbative regime (bottom row), TPR=1 in all except four trajectories in Example 1 and one trajectory in Example 3, yet these learned trajectories still capture ${\mathcal {H}}^{\mu }_0$ accurately (see Fig. 9).

#### Pointwise agreement with ${ {\mathcal {H}}^{\mu }_0}$ (Fig. 9)

Figure 9 displays $\Delta H$ values for each learned trajectory (48) from which we observe excellent qualitative agreement with the ${\mathcal {H}}^{\mu }_0$ dynamics across all trials. This indicates that the level curves of $\widehat{{\mathcal {H}}}^\mu _0$ are very close to those of ${\mathcal {H}}^{\mu }_0$ despite having been identified using a data sampled from a single level curve. The numerical range of $\Delta H$ corresponding to the color gradations in Fig. 9 is provided in Table 2. For the mild perturbative regime (top row), the method produces a maximum $\Delta H$ of 0.012 (with the exception of $\Delta H = 0.087$ on the inner-most trajectory of Example 1), and a the maximum of $\Delta H = 0.053$ over the extreme regime (bottom row). To summarize, given a single trajectory from $H_\varepsilon$, the learned Hamiltonian $\widehat{{\mathcal {H}}}^\mu _0$ using WSINDy agrees with the analytical reduced Hamiltonian ${\mathcal {H}}^{\mu }_0$ over a large region of phase space, and with very mild dependence on the perturbative regime: $\Delta H$ is upper-bounded by $\varepsilon$ in almost all cases.

**Table 2:** Range of $\Delta H$ values across initial conditions for each example.

	Ex1	Ex2	Ex3	Ex4
(min $\Delta H$, max $\Delta H$), mild	(0.003, 0.012), outlier 0.087	(0.004, 0.006)	(0.003, 0.012)	(0.006, 0.018)
(min $\Delta H$, max $\Delta H$), extreme	(0.014, 0.053)	(0.021, 0.033)	(0.012, 0.035)	(0.017, 0.044)

**Table 3:** Range of forward simulation errors across initial conditions for each example.

	Ex1	Ex2	Ex3	Ex4
(min $\Delta \textbf{Z}^\mu$, max $\Delta \textbf{Z}^\mu$), mild	(0.004, 0.082)	(0.007, 0.015)	(0.001, 0.022)	(0.036, 0.419)
(min $\Delta \textbf{Z}^\mu$, max $\Delta \textbf{Z}^\mu$), extreme	(0.015, 0.315)	(0.024, 0.044)	(0.005, 0.128)	(0.043, 0.435)
(min $\Delta \textbf{Z}$, max $\Delta \textbf{Z}$), mild	(0.015, 0.124)	(0.056, 0.064)	(0.005, 0.018)	(0.031, 0.314)
(min $\Delta \textbf{Z}$, max $\Delta \textbf{Z}$), extreme	(0.069, 0.579)	(0.065, 0.067)	(0.019, 0.145)	(0.050, 0.468)
(min $\Delta \textbf{Z}^*$, max $\Delta \textbf{Z}^*$), mild	(0.015, 0.084)	(0.058, 0.065)	(0.005, 0.019)	(0.015, 0.238)
(min $\Delta \textbf{Z}^*$, max $\Delta \textbf{Z}^*$), extreme	(0.067, 0.409)	(0.071, 0.080)	(0.018, 0.068)	(0.082, 0.408)

#### Forward simulation errors (Table 3)

In Table 3 we report forward simulation errors according to $\Delta \textbf{Z}^\mu$ and $\Delta \textbf{Z}$, with $\Delta \textbf{Z}^\star$ as a reference (see (49)–(51)). See Figures S5 and S8 in the Supplemental Information for similar visualizations to Figs. 8 and 9. In the mild regime, Examples 1-3 yield ${\mathcal {O}}(10^{-2})$ errors for both $\Delta \textbf{Z}^\mu$ (agreement with ${\mathcal {H}}^{\mu }_0$) and $\Delta \textbf{Z}$ (agreement with $H_\varepsilon$). Example 4 exhibits much larger forward simulation errors due to the chaotic nature of the dynamics, rendering $\Delta \textbf{Z}$ and $\Delta \textbf{Z}^\mu$ unreliable since $\Delta H = {\mathcal {O}}(10^{-2})$ and TPR=1 in all trials.

In the extreme perturbative regime, the method produces accurate forward simulations for Examples 2 and 3 according to both $\Delta \textbf{Z}^\mu$ and $\Delta \textbf{Z}$, with exceptional agreement on Example 2, providing an improvement over the analytical reduced Hamiltonian ${\mathcal {H}}^{\mu }_0$ (i.e. $\Delta \textbf{Z}< \Delta \textbf{Z}^\star$). This is especially surprising because Example 2 exhibits strong mixing between the slow and fast timescales (see Fig. 5). Larger values of $\Delta \textbf{Z}^\mu$ and $\Delta \textbf{Z}$ are observed for Examples 1 and 4, despite small $\Delta H$ values. For Example 1 this is due to minor phase differences which rapidly accumulate errors, while Example 4 suffers again because of its chaotic nature.

### Results: nonzero extrinsic noise ($\sigma _{NR}>0$)

We now survey the performance of WSINDy in recovering ${\mathcal {H}}^{\mu }_0$ from data with extrinsic (measurement) noise. For each example we take a fixed trajectory from the extreme perturbative regime for which WSINDy recovers the correct model (TPR=1) when $\sigma _{NR}=0$, and we add various levels of Gaussian white noise. Results are averaged over 100 independent instantiations of noise. Figures S10-S13 of the Supplemental Information provide visualizations of recovery at $10\%$ noise ($\sigma _{NR}=0.1$).

**Figure 10:**
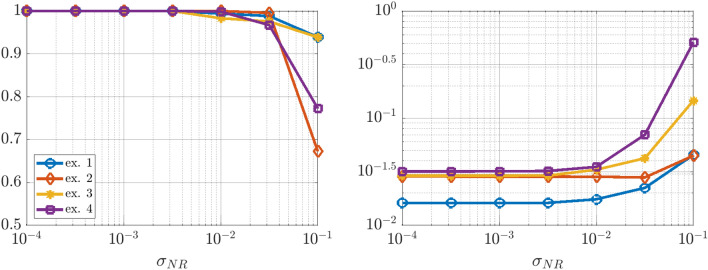
Results with noisy data. Left: TPR, right: $\Delta H$. Results averaged over 100 trials.

Average TPR and $\Delta H$ values at noise levels $\sigma _{NR}\in [10^{-4},10^{-1}]$ (see (43)) are presented in Fig. 10 (see also Figure S9 of the Supplemental Information for similar assessments of $\Delta \textbf{Z}^\mu$ and $\Delta \textbf{Z}$). From Fig. 10 we observe similar trends across all examples in the low-medium noise regime. The method is robust up until $\sigma _{NR} \approx 10^{-1.5}\approx 3\%$ noise, providing on average $\text {TPR}>0.95$ and $\Delta H<0.04$.

At $10\%$ noise ($\sigma _{NR} = 0.1$), WSINDy is still able to recover the model structure of Examples 1 and 3 very well (TPR$\approx 0.95$), yet for Example 3 this coincides $\Delta H>10\%$. Example 2 exhibits the lowerest TPR at $10\%$ noise, but this coincides with $\Delta H< 4\%$ on average, indicating identification of a different reduced Hamiltonian that still agrees well with ${\mathcal {H}}^{\mu }_0$. Moreover, Figure S9 of the Supplemental Information indicates that on average these learned model perform as well as the analytical reduced dynamics given by ${\mathcal {H}}^{\mu }_0$, according to $\Delta \textbf{Z}$. Since ${\mathcal {H}}^{\mu }_0$ is only a leading-order approximation, a natural next line of inquiry is to examine the connection between “misspecified” models and analytical higher-order corrections.

For Example 4, 10% noise is clearly outside of the feasible recovery regime, with misspecified models (TPR<1) leading to large values of $\Delta H$. This can be overcome by considering multiple trajectories (not shown here), which serves to recover $\text {TPR}=1$, yet accuracy issues with $\Delta H$ remain. We conjecture that some form of variance reduction is needed. We aim to investigate the applicability of WENDy (Weak-form estimation of nonlinear dynamics)^18^ which has been demonstrated to reduce regression errors due to extrinsic noise.

The combined effects of extrinsic noise and large intrinsic perturbations represent a significant challenge, and the results in Fig. 10 improve greatly under less severe corruption levels. Still, under these adverse conditions WSINDy is able to identify coarse-grained models with adequate performance across examples, suggesting that the method is well-suited for scientific discovery with these mixed effects.

## Conclusions

In this article we have described a weak-form equation learning approach to coarse-graining Hamiltonian systems using the WSINDy algorithm. We have provided substantial evidence that appropriate coarse-grained models with Hamiltonian structure can be identified from a single noisy trajectory simply by choosing a proper weak formulation. The resulting coarse-grained models have the potential to be very useful surrogate models in forward simulations. Moreover, level sets of the reduced Hamiltonian are readily captured, proving global information about the energy landscape. This is significant due to the nontrivial analytical procedures involved in deriving reduced Hamiltonian systems using nearly-periodic reduction techniques. The dictionary learning approach naturally enforces structure preservation, and the method is highly efficient, requiring no forward solves of candidate models. The output is a human-readable equation for the reduced-order Hamiltonian which can then be used in down-stream tasks.

The fact that a suitable weak formulation enables one to identify the reduced-order Hamiltonian over all of phase space using only a single (noisy) trajectory is surprising, and to the best of the authors’ knowledge not found in the literature. An obvious next direction is to leverage this in combination with black-box methods (neural networks, Gaussian processes, etc.), and to adapt the test functions to target different models in a hierarchical fashion, as demonstrated in Figs. 1 and 2. We aim also in a future work to quantify the sufficiency of information needed to identify the reduced Hamiltonian, as results from degenerate cases (trajectories that only encircle one of the relevant fixed points, see Examples 1 in Fig. 8) imply that the reduced Hamiltonian is accessible from regions of phase with seemingly low levels of information. At the other extreme, combining information from multiple trajectories (as typically done in black-box learning approaches) is an obvious means of increasing performance. Answering questions of data and information sufficiency will be crucial to developing methods of identifying higher-order (in $\varepsilon$) coarse-grained models.

Finally, combined with findings in^31^, a major implication of our results is that the weak form opens the door to a new generation of coarse-graining algorithms. We aim to explore the use of these algorithms in surrogate modeling to reduce the number of full simulations of dynamics involving disparate scales, which are computationally prohibitive.

### Supplementary Information

Supplementary Information.

## Data Availability

The datasets and code used in the present work are publicly available at https://github.com/MathBioCU/WSINDy_H. Please contact the corresponding author (DAM) with any additional inquiries.
