# Supplementary material for: Coarse-graining Hamiltonian systems using WSINDy
Source: Sci Rep. 2024 Jun 24;14:14457. doi: 10.1038/s41598-024-64730-0 (PMC11196701; doi:10.1038/s41598-024-64730-0)
Supplement: Supplementary file 1 — Supplementary Information. [file 41598_2024_64730_MOESM1_ESM.pdf]

Coarse-Graining Hamiltonian Systems Using WSINDy: Supplemental Information

Daniel A. Messenger[1,*], Joshua W. Burby[2,†], and David M. Bortz[1,+]

[1]University of Colorado, Department of Applied Mathematics, Boulder, CO, 80309-0526, USA
[2]Los Alamos National Laboratory, Theoretical Division, Los Alamos, NM, 87545, USA
[*]daniel.messenger@colorado.edu
[†]jburby@lanl.gov
[+]david.bortz@colorado.edu

S1 Robust cornerpoint method

In [1], the authors developed a method of choosing the test function hyperparameters using a cornerpoint algorithm applied to the Fourier spectrum of the data. This approach was taylored to the case of extrinsic noise, in which case the cumulative sum of the power spectrum of the data can be approximated be two line segments, the intersection of which (the cornerpoint) closely marks the changepoint from signal-dominated to noise-dominated Fourier modes. For nearly periodic systems without extrinsic noise, the two line segment assumption is not accurate, however, a feasible test functions support width T_ϕ can still be computed using a slightly altered cornerpoint approach, simply by taking the minimum of the rotated cumulative sum of the power spectrum. We also extend the method in [1] to work with any test function, not just those approximated by Gaussian distributions. Section S1.2 contains a qualitative analysis of the dependence of Hamiltonian coarse-graining results on T_ϕ. Figure S2 shows that the method proposed here is highly successful at finding T_ϕ that lie in the feasible recovery region (yielding TPR=1).

S1.1 Method overview

Denote the discrete Fourier transform (DFT) of the nth component of the data $\mathbf{Z}$ by

$$\mathcal{F}[\mathbf{Z}_n](k) := \sum_{j=0}^{m-1} \exp\left(-\frac{2\pi i}{m} jk\right) \mathbf{Z}_n(t_j)$$

and let its cumulative sum (taken over the negative wavenumbers) be denoted by

$$\mathbf{H}_n(k) = \sum_{k'=-m/2}^{k} \left|\mathcal{F}[\mathbf{Z}_n](k')\right|, \quad k \in \{-m/2, \dots, 0\}.$$

For convenience we will assume m is even as the odd case is nearly identical. With high probability, $\mathbf{H}_n$ will lie below the line

$$k \to \mathbf{Y}_n(k) := \left(\frac{k_{\max} - k}{k_{\max} + (m/2)}\right) \mathbf{H}_n(-m/2) + \left(\frac{k + (m/2)}{k_{\max} + (m/2)}\right) \mathbf{H}_n(k_{\max})$$

where $k_{\max} = \operatorname{argmax}_{k \in \{-m/2,\dots,0\}} |\mathcal{F}[\mathbf{Z}_n](k)|$ is the wavenumber of the largest Fourier coefficient. A tie is broken by the maximizing k with the largest magnitude. Moreover, $\mathbf{H}_n$ will be approximately convex for $k \in \{-m/2, \dots, k_{\max}\}$. For instance, convexity and $\mathbf{H}_n \leq \mathbf{Y}$ over this region are both true if $\mathcal{F}[\mathbf{Z}_n]$ exhibits any decay of the form $|\mathcal{F}[\mathbf{Z}_n](k)| \sim |k|^{-s}$ for $s > 0$. We define the cornerpoint of $\mathbf{H}_n$ as the point $(k_n^*, \mathbf{H}_n(k_n^*))$ that maximizes the distance between $\mathbf{H}_n(k)$ and $\mathbf{Y}_n(k)$ over $k \in \{-m/2, \dots, k_{\max}\}$ in the total least squares sense. A practical computation of this is the following. Let $R(\theta)$ be the rotation matrix in the $(k, \mathbf{H}_n)$ plane such that

$$\begin{pmatrix} 0 \\ 1 \end{pmatrix} \cdot \mathbf{R}(\theta) \begin{pmatrix} k \\ \mathbf{Y}_n(k) \end{pmatrix} = 0.$$

That is, $\mathbf{R}(\theta)$ rotates $\mathbf{Y}_n$ parallel to the k-axis. Then

$$k_n^* := \operatorname*{argmin}_{k \in \{-m/2,\dots,k_{\max}\}} \left[\mathbf{R}(\theta) \begin{pmatrix} k \\ \mathbf{H}_n(k) \end{pmatrix}\right]_2$$

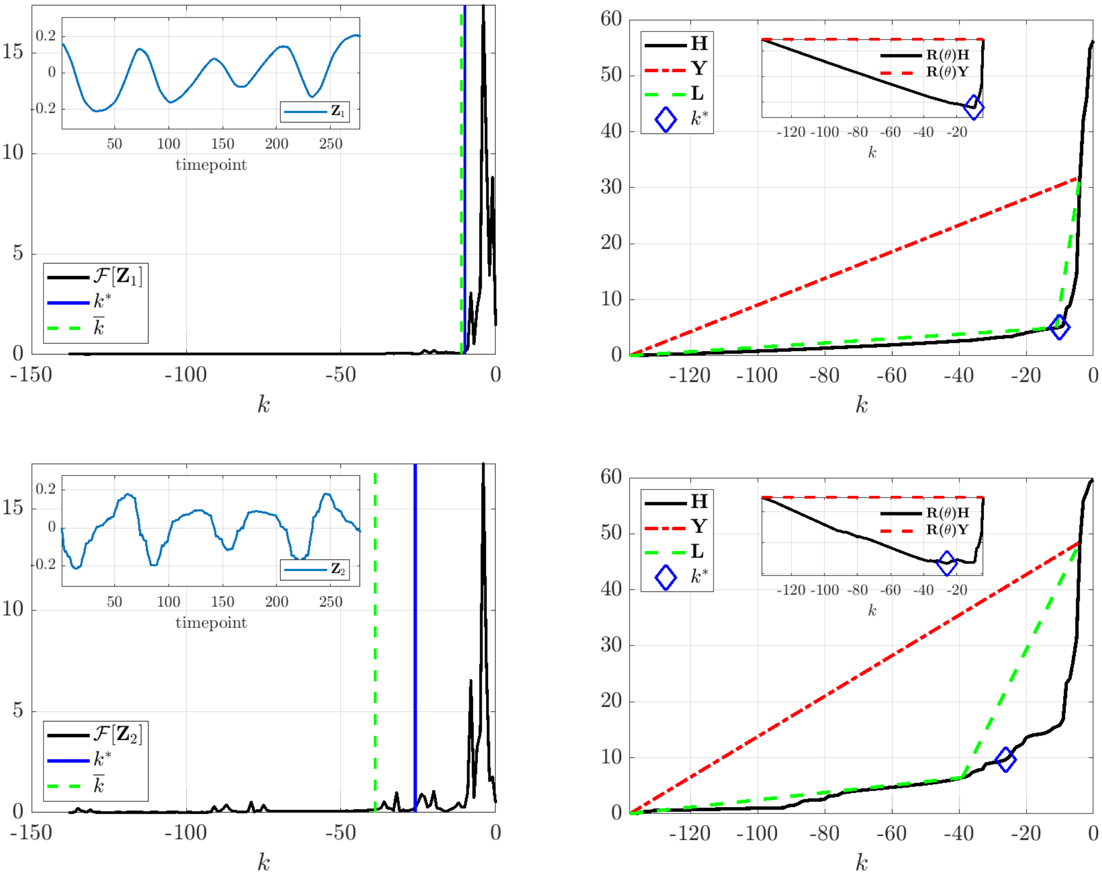

Figure S1. Visualization of cornerpoint detection and comparison to the method used in [1]. The data $\mathbf{Z}$ is taken from Example 2 with $\varepsilon = 0.05$ and $Q(0) = \frac{31\pi}{32}$. The top and bottom rows display coordinates 1 and 2, the configuration and momentum variables for the first oscillator. The data and DFTs are plotted in the left column, along with markers for the detected cornerpoints, while the right column shows the cumulative sums $\mathbf{H}_n$ together with the line $\mathbf{Y}$ and the piecewise linear approxmation $\mathbf{L}$ used in [1] (inset plots show the rotated $\mathbf{H}$ which is minimized at k^*). The value k^* is the cornerpoint detected by the current method, while $\bar{k}$ is the value detected by the method in [1]. In the top row, the two methods nearly agree, while the more perturbed dynamics in the bottom row cause the $\bar{k}$ to be significantly larger, leading to more irrelevant modes entering the recovered model.

where $[\cdot]_2$ indicates the second component of the vector. This process is visualized in Figure S1, and in this work, we let the final cornerpoint be the average $k^* = \frac{1}{2N}\sum_{n=1}^{2N} k_n^*$, used for all observed variables. Figure S1 displays that the previous method is more prone to labeling irrelevant modes as signal-dominated (2nd row), but overall the performance of both methods is comparable.

S1.2 Dependence of Hamiltonian coarse-graining on T_ϕ (Figure S2)

As seen in Section 4.1 of the main text, the ratio between the test function support width T_ϕ and the fast scale T_f, $\sigma_{\phi f} = T_\phi / T_f$, plays a role in the accuracy of $\widehat{\mathcal{H}}_0^\mu$ with respect to $\mathcal{H}_0^\mu$. This relationship is graphically represented here in Figure S2, where we present the TPR, ΔH, and $\Delta \mathbf{Z}^\mu$ values (equations (47)-(51)) for Examples 1-4 over a range of $\sigma_{\phi f}$, as well as the $\sigma_{\phi f}$ values resulting from T_ϕ as computed using the method described above (black curves). In Figure S2 we display only the results for the extreme perturbative regime (see Section 5.5 of the main text) as the method performs very well for a wide range of $\sigma_{\phi f}$ in the mild regime.

Several general trends can be observed when $\sigma_{\phi f}$ is varied. From the perspective of the TPR score, it can been seen that the optimal T_ϕ is usually associated with some $\sigma_{\phi f} \in [5, 30]$, and the exact optimal $\sigma_{\phi f}$ is highly dependent on the trajectory. Comparing the first and second columns, we see that TPR=1 always correlates with lower ΔH, yet there are instances where the

Hamiltonian is captured accurately (low ΔH) without correct identification of $\mathcal{H}_0^\mu$ (e.g. Example 3, $z(0)$ index 10). On the other hand, an accurate forward solve does not always correlate with TPR, so from the perspective of $\Delta \mathbf{Z}^\mu$ we get a different optimal $\sigma_{\phi f}$. Often larger $\sigma_{\phi f}$ will yield lower forward simulation errors $\Delta \mathbf{Z}^\mu$ despite not capturing the correct form of $\mathcal{H}_0^\mu$ (i.e. TPR<1). This is most readily observed from Examples 3 and 4 (right column), as well as Example 1 for $z(0)$ indices $6-10$. This serves to highlight the importance of examining multiple performance metrics, as the down-stream task (forward simulations, scientific inference, etc.) should determine which metric is weighted most highly.

The black curve indicates values of $\sigma_{\phi f}$ (hence T_ϕ) resulting from default settings of the method as presented here. In all cases except $z(0)$ indices $\{8,9,10,12\}$ of Example 1 and $z(0)$ index 10 in Example 3, the identified T_ϕ yields the correct model terms, which automatically grants excellent agreement with $\mathcal{H}_0^\mu$ (column 2). In these five cases with TPR < 1, the Hamiltonian is still very accurate, with $\Delta H \approx 0.02$. For Example 2, the black curve successfully lies in the region of admissible $\sigma_{\phi f}$ values, which is narrow due to mixing of slow and fast scales which causes lower ΔH and $\Delta \mathbf{Z}^\mu$ at larger T_ϕ. Results are similar for Example 3. In Example 4 the correct model is found for nearly all $\sigma_{\phi f}$ and $z(0)$, although larger T_ϕ may increase accuracy.

S2 MSTLS algorithm

To solve for a sparse coefficient vector $\widehat{\mathbf{w}}$ such that $\mathbf{G}\widehat{\mathbf{w}} \approx \mathbf{b}$, we use the MSTLS algorithm proposed in [1] that uses the original STLS algorithm from [2] with variable thresholding and then performs a line search for the sparsity threshold λ. By heterogeneous thresholding, we mean that instead of employing the typical hard thresholding operator

$$H_\lambda(\mathbf{w})_j = \begin{cases} \mathbf{w}_j, & |\mathbf{w}_j| \geq \lambda \\ 0, & \text{otherwise,} \end{cases} \tag{S1}$$

which treats all columns of $\mathbf{G}$ equally, we define the variable hard-thresholding operator

$$H_{\mathbf{L},\mathbf{U}}(\mathbf{w})_j = \begin{cases} \mathbf{w}_j, & L_j \leq |\mathbf{w}_j| \leq U_j \\ 0, & \text{otherwise} \end{cases} \tag{S2}$$

for specified upper and lower bound vectors $\mathbf{U}, \mathbf{L} \in \mathbb{R}^J$ (for a J-term library $\mathbb{H}$). This is particular useful for enforcing not only that the coefficients $\mathbf{w}_j$ stay within a certain range, but also that the term magnitudes $\|\mathbf{G}_j \mathbf{w}_j\|$ have a reasonable contribution to the dynamics given by $\mathbf{b}$.

In this work, we define $\mathbf{L}$ and $\mathbf{U}$ to enforce that $\widehat{\mathbf{w}}_j$ and $\widehat{\mathbf{w}}_j \mathbf{G}_j$ are comparable to the best 1-term solution. That is, denoting the projection operator by $\mathbf{P}$, we define

$$\hat{j} := \underset{j}{\operatorname{argmax}} \|\mathbf{P}_{\mathbf{G}_j} \mathbf{b}\|_2, \qquad \hat{w} := \frac{|\mathbf{b} \cdot \mathbf{G}_{\hat{j}}|}{\|\mathbf{G}_{\hat{j}}\|_2^2}, \qquad \hat{G} := \frac{\hat{w}\|\mathbf{G}_{\hat{j}}\|_2}{\|\mathbf{b}\|_2}$$

where $\hat{w}$ and $\hat{G}$ are the coefficient and relative term magnitudes of the best 1-term projection of $\mathbf{b}$ onto the library $\mathbb{H}$. We then let

$$L_j(\lambda) = \lambda^{-1} \max\{\hat{w}, \hat{G}/\|\mathbf{G}_j\|_2\} \tag{S3}$$

$$U_j(\lambda) = \lambda \min\{\hat{w}, \hat{G}/\|\mathbf{G}_j\|_2\} \tag{S4}$$

which implies that the nonzero entries of $H_{\mathbf{L}(\lambda),\mathbf{U}(\lambda)}(\mathbf{w})$ are the entries of $\mathbf{w}$ satisfying

$$\lambda \hat{w} \leq |\mathbf{w}_j| \leq \lambda^{-1} \hat{w} \qquad \& \qquad \lambda \hat{G} \leq \frac{|\mathbf{w}_j| \|\mathbf{G}_j\|_2}{\|\mathbf{b}\|_2} \leq \lambda^{-1} \hat{G}.$$

The MSTLS algorithm then proceeds as follows. With initial guess $\mathbf{w}^{(0)} = \mathbf{G}^\dagger \mathbf{b}$ and support set $S^{(0)} = \{1, \dots, J\}$ the inner STLS loop for fixed λ is defined by

$$(\text{STLS}) \qquad \begin{cases} S^{(\ell+1)} = \operatorname{supp}\left(H_{\mathbf{U}(\lambda),\mathbf{L}(\lambda)}(\mathbf{w}^{(\ell)})\right) \\ \mathbf{w}^{(\ell+1)} = \underset{\operatorname{supp}(\mathbf{w}) \subset S^{(\ell+1)}}{\operatorname{argmin}} \|\mathbf{G}\mathbf{w} - \mathbf{b}\|_2^2 \end{cases} \tag{S5}$$

which is run until termination, which must occur in maximum J iterations [3]. Denoting the solution by $\text{STLS}(\mathbf{G}, \mathbf{b}; \lambda) = \mathbf{w}^\lambda$ (where $\mathbf{w}^0 = \mathbf{w}^{(0)}$), we then define the auxiliary loss function $\mathcal{L}$ by

$$\mathcal{L}(\lambda) = \frac{\|\mathbf{G}(\mathbf{w}^\lambda - \mathbf{w}^0)\|_2}{\|\mathbf{G}\mathbf{w}^0\|_2} + \frac{\|\mathbf{w}^\lambda\|_0}{\|\mathbf{w}^0\|_0}, \quad \mathbf{w}^\lambda = \text{STLS}(\mathbf{G}, \mathbf{b}; \lambda). \tag{S6}$$

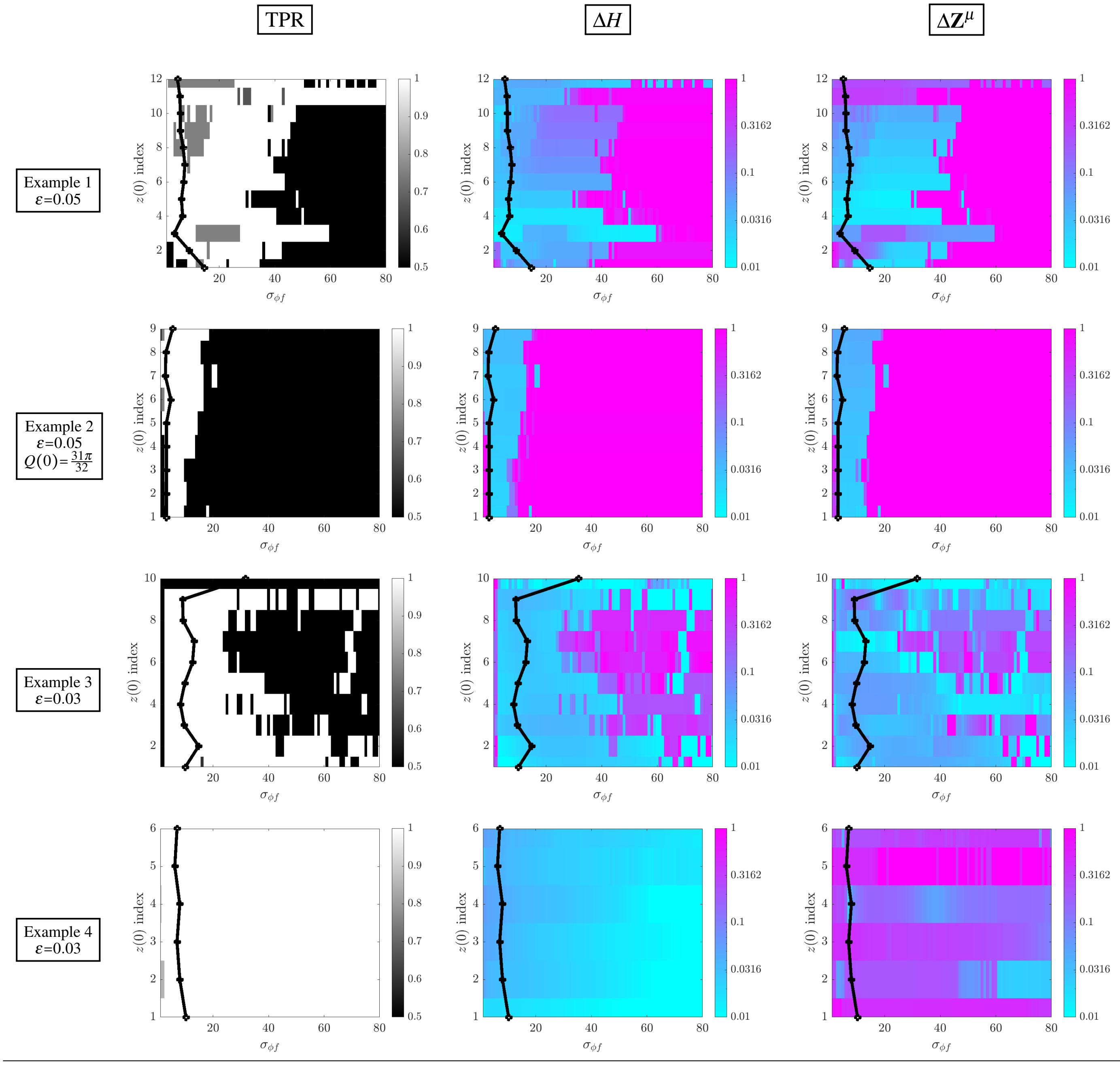

Figure S2. Dependence of Hamiltonian coarse-graining on test function radius. Values of the TPR, ΔH, and $\Delta \mathbf{Z}^{\mu}$ (see eqs. (47)-(51)) statistics in the extreme perturbative regime as a function of the ratio $\sigma_{\phi f} = T_{\phi}/T_f$ (see eq. (27)) and the learning trajectory (indicated on the y-axes as the $z(0)$ index). Black curves indicates values of $\sigma_{\phi f}$ resulting from using default settings of the Robust Corner Point method to find T_{ϕ}.

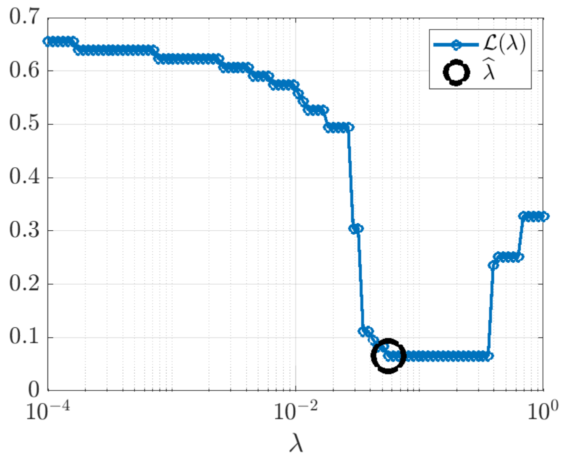

Figure S3. Example profile of the loss function $\mathcal{L}(\lambda)$ employed in MSTLS (S7). Data taken from Figure S10.

Finally, we define MSTLS with candidate sparsity thresholds $\boldsymbol{\lambda}$ by

$$\text{(MSTLS)} \qquad \begin{cases} \widehat{\lambda} = \min\left\{\lambda \in \boldsymbol{\lambda} \ : \ \mathcal{L}(\lambda) = \min_{\lambda \in \boldsymbol{\lambda}} \mathcal{L}(\lambda)\right\} \\ \widehat{\mathbf{w}} = \text{STLS}(\mathbf{G}, \mathbf{b}; \widehat{\lambda}). \end{cases} \tag{S7}$$

We denote by $\text{MSTLS}(\mathbf{G}, \mathbf{b}, \boldsymbol{\lambda}) := \widehat{\mathbf{w}}$ the output weight vector in (S7). The collection of thresholds $\boldsymbol{\lambda}$ can be chosen by the user. Following the strategy seen to be successful in [1], we let $\boldsymbol{\lambda}$ be 100 equally log-spaced values from 10^{-4} to 1. This process is visualized in Figure S3.

S3 Initial conditions for forward simulations

In order to measure agreement between forward simulations of $\mathcal{H}_0^\mu$ and $\widehat{\mathcal{H}}_0^\mu$, we first use a data-driven nonlinear least squares approach to find an adequate set of initial conditions. For all time intervals $I_k = [t_0, t_k]$, $k = 2, \dots, 100$, we fit a polynomial of degree 2 through the data $\mathbf{Z}(I_k)$ and let $z(0)$ be the value of this polynomail at t_0. We then simulate the system of interest (defined by $\mathcal{H}_0^\mu$ or $\widehat{\mathcal{H}}_0^\mu$) starting from the selected $z(0)$ for 1/8 of the total time of the available data. The initial condition $z(0)$ that minimizes the error between these partial forward simulations and the data $\mathbf{Z}$ over the 99 time intervals I_k is chosen for forward simulations.

S4 Supplemental Information for Section 4.1

Figure S4 is complementary to Figure 1 of the main text, showing that the three models identified by WSINDy using different values of T_ϕ are accurate dynamically as well as over phase space.

S5 Supplemental Information for Section 5.8: Forward Simulation Accuracy

Figures S5-S8 provide forward simulation accuracy results and visualization to complement Table 3 in the main text. The right columns of Figures S5-S8 display $\Delta\mathbf{Z}^\mu$ and $\Delta\mathbf{Z}$ for the extreme perturbative regime, where larger values of $\Delta\mathbf{Z}^\mu$ and $\Delta\mathbf{Z}$ can be observed for Examples 1 and 4 (Figures S5 and S8), while Examples 2 and 3 accurately capture both the reduced dynamics of $\mathcal{H}_0^\mu$ and the full dynamics of H_ε. As a reference, the bottom rows of Figures S5-S8 plot simulations from $\mathcal{H}_0^\mu$, the analytical reduced Hamiltonian, colored according to $\Delta\mathbf{Z}^\star$, for comparison with $\Delta\mathbf{Z}$.

Forward simulation accuracy results for noisy data are shown in Figure S9. For Example 1 (blue curves), accuracy is mildly dependent on noise, remaining close to the $\mathcal{H}_0^\mu$ dynamics (left plot) while $\varepsilon = 0.05$ imparts large errors with respect to the full dynamics governed by H_ε (as measured by $\Delta\mathbf{Z}$, right plot) due to phase shifts which persist in the analytical reduced dynamics give by $\mathcal{H}_0^\mu$ (see Figure S10, right). In Example 2, results are also only mildly noise dependent, and even in the presence of noise the learned models improve on the analytical reduced dynamics, as the black dashed line in the right plot (denoting $\Delta\mathbf{Z}^\star$) lies above the red curve, which shows the trend in $\Delta\mathbf{Z}$. At low noise levels, Example 3 produces dynamics that agree well

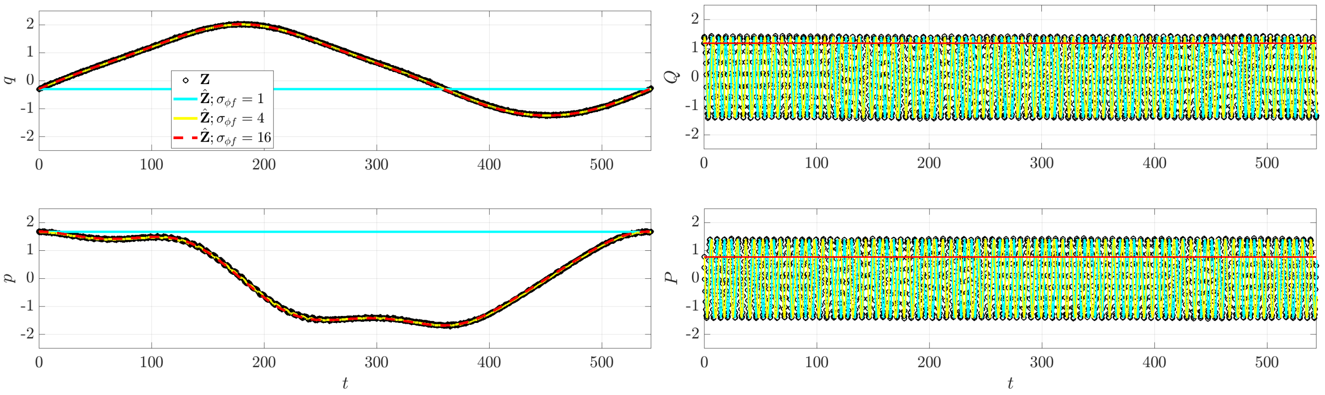

Figure S4. Multi-model inference viewed dynamically. Time series plots of the data and learned systems from Figure 1 of the main text. The coarse-grained model (red) accurately captures the slow dynamics (left, yellow), while the fast-scale model (cyan) accurately captures the fast dynamics.

with $\mathcal{H}_0^\mu$, but which impart significant errors at high noise due to phase shifts. Learned models from Example 4 do not remain close to the $\mathcal{H}_0^\mu$ or H_ε dynamics due to chaos. Since the electric potential φ is not bounded, the two particles eventual wander through the plane, visiting different lattice sights (see Figure S13).

S6 Supplemental Information for Section 5.9: Visualizing noisy data

Figures S10-S13 visualize model recovery from data with 10% noise for Examples 1-4, with the noisy data $\mathbf{Z}$ in red, the clean data $\mathbf{Z}^\star$ in black, the true reduced data $\mathbf{Z}^\mu$ in blue, and the learned reduced data $\widehat{\mathbf{Z}}^\mu$ in green. Figure S10, phase shifts in the learned and analytical reduced dynamics can be seen for Example 1, both in agreement with each other. We leave investigation of corrections to such phase shifts, and their relation to higher-order corrections, to future work.

However, Example 1 indicates that capturing the phase of a slow oscillator for arbitrarily many periods may require additional constraints on the model.

References

1. Messenger, D. A. & Bortz, D. M. Weak SINDy For Partial Differential Equations. J. Comput. Phys. **443**, 110525, DOI: 10.1016/j.jcp.2021.110525 (2021).
2. Brunton, S. L., Proctor, J. L. & Kutz, J. N. Discovering governing equations from data by sparse identification of nonlinear dynamical systems. Proc. Natl. Acad. Sci. **113**, 3932–3937, DOI: 10.1073/pnas.1517384113 (2016).
3. Zhang, L. & Schaeffer, H. On the Convergence of the SINDy Algorithm. Multiscale Model. Simul. **17**, 948–972, DOI: 10.1137/18M1189828 (2019).

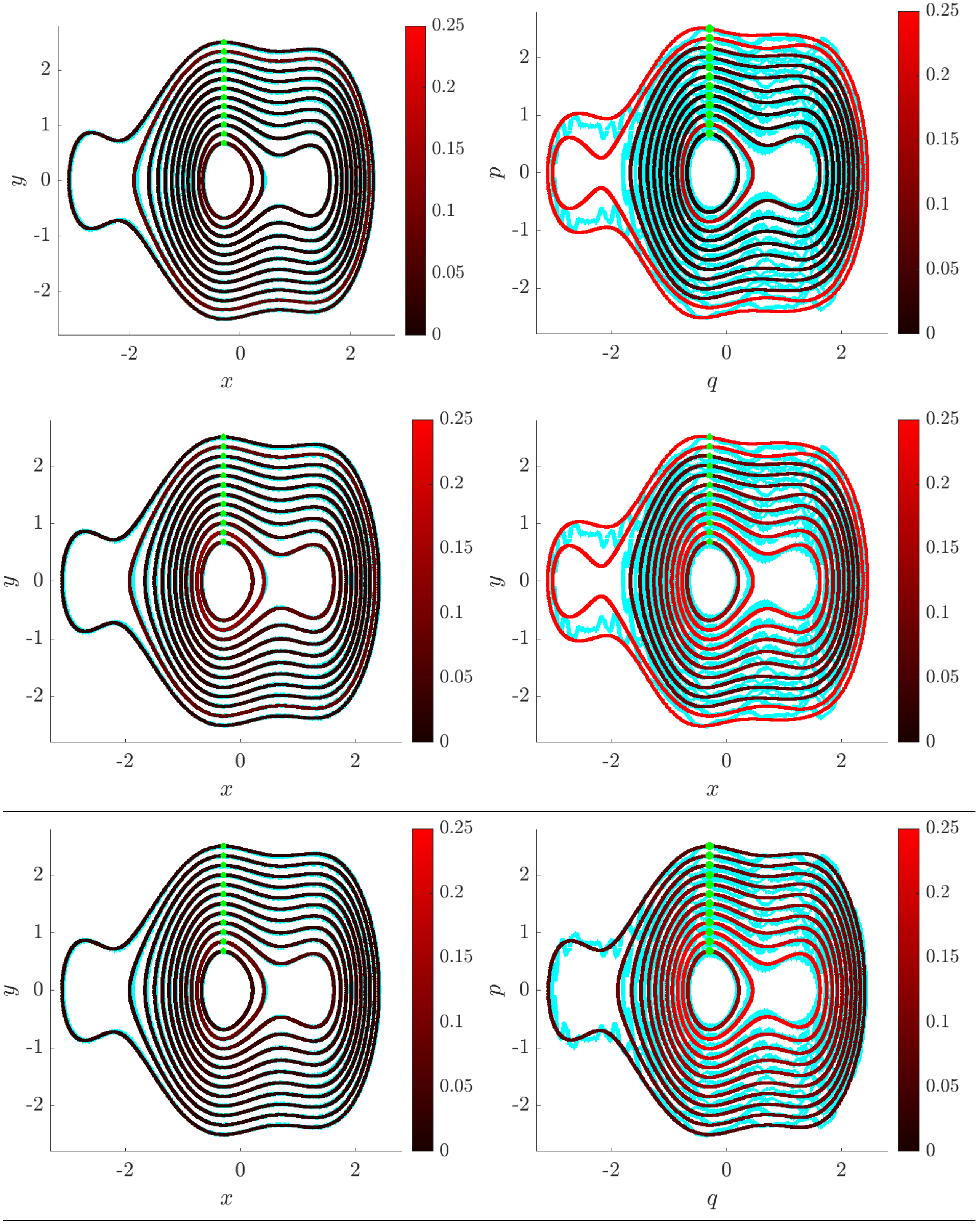

Figure S5. Forward simulation accuracy for Example 1. Top to bottom: red-scale trajectories colored according to $\Delta\mathbf{Z}^{\mu}$, $\Delta\mathbf{Z}$, and $\Delta\mathbf{Z}^{\star}$ (defined in (47)-(51) of the main text). In the top and middle rows, trajectories are simulated from the learned Hamiltonian $\widehat{\mathcal{H}}_0^{\mu}$, while in the bottom row they are simulated from $\mathcal{H}_0^{\mu}$. The training data is plotted in cyan. Green dots indicate initial conditions. Left and right columns show results for the mild and perturbative regimes (see Section 5.5 for details).

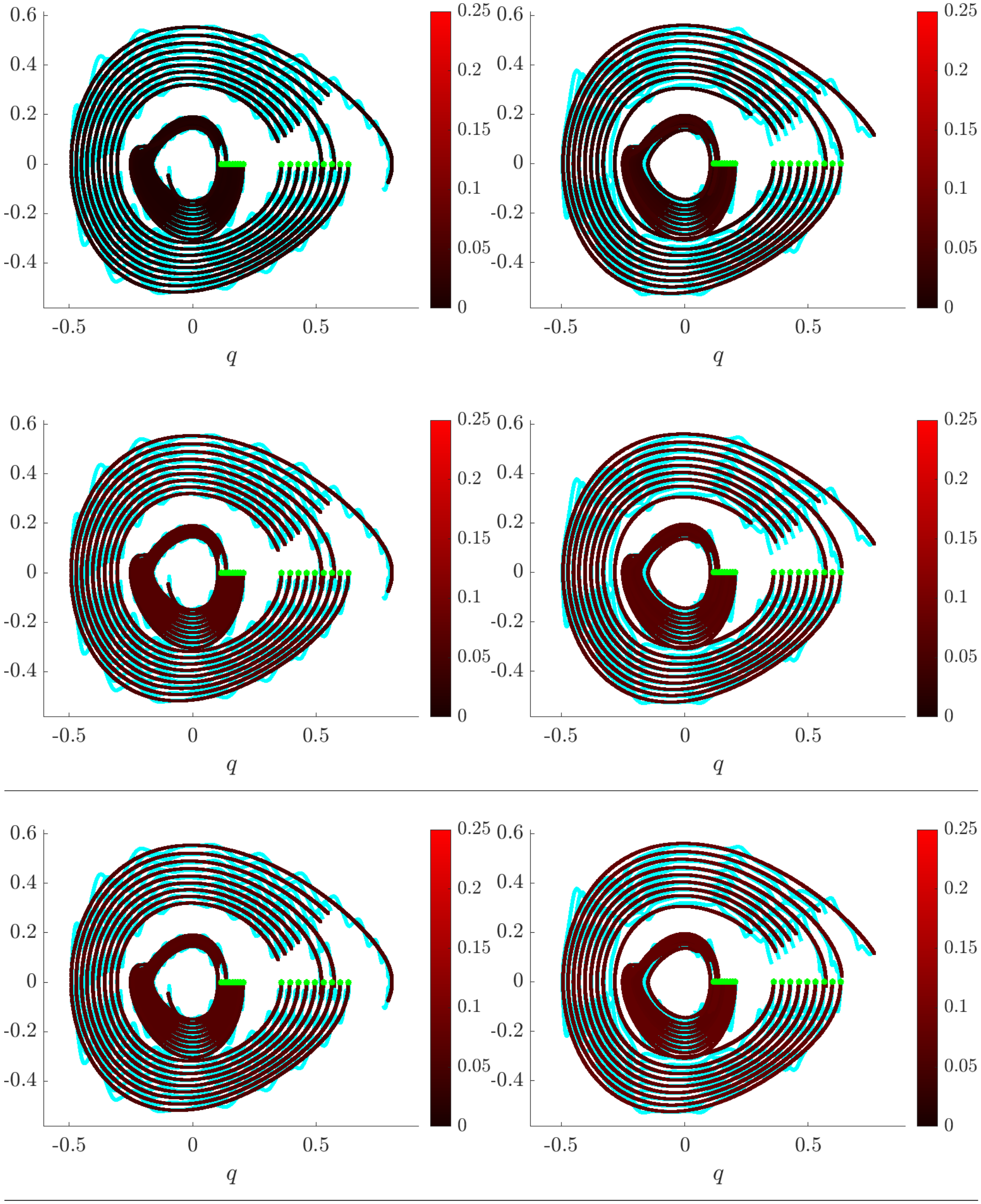

Figure S6. Forward simulation accuracy for Example 2. Same layout as Figure S5.

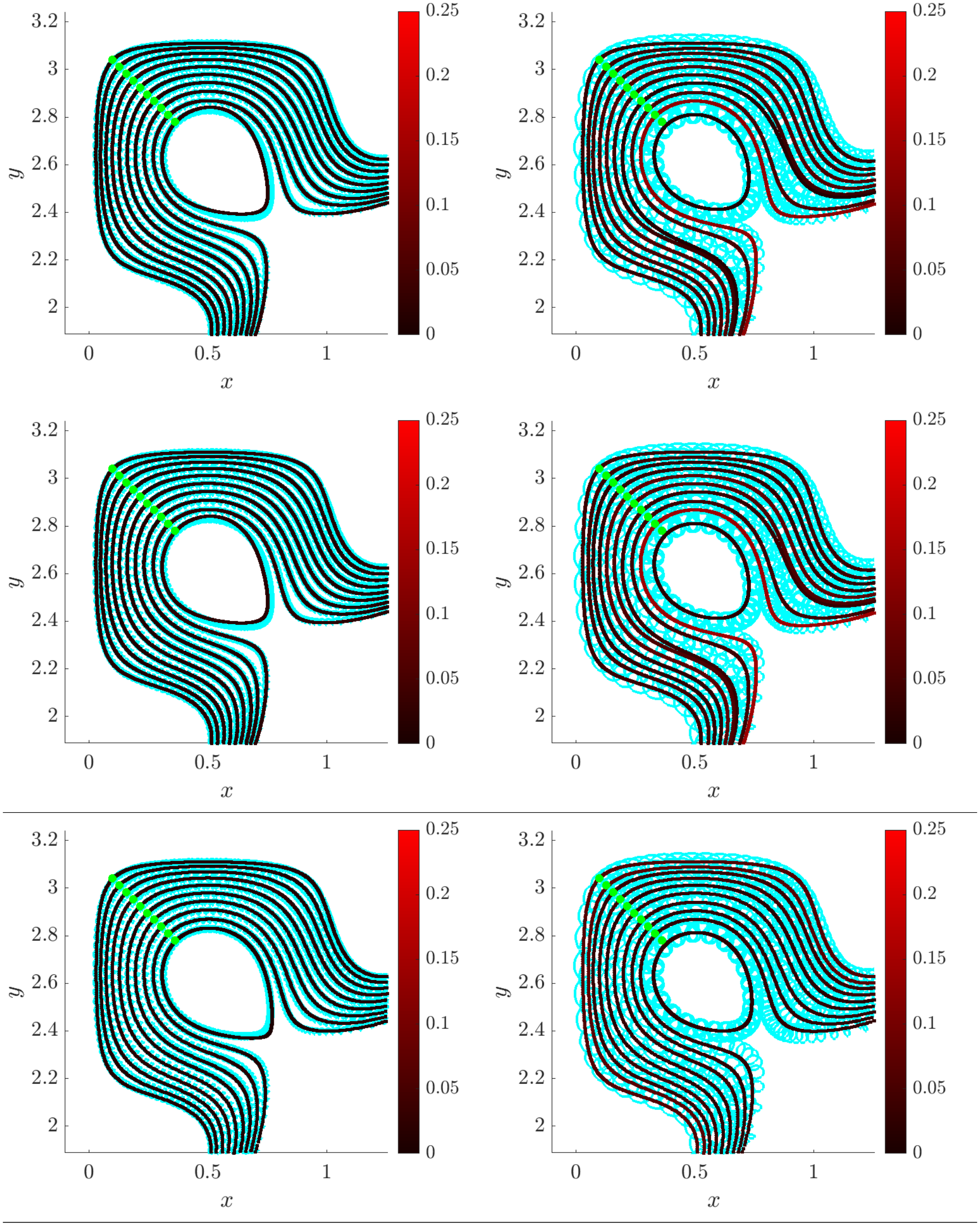

Figure S7. Forward simulation accuracy for Example 3. Same layout as Figure S5.

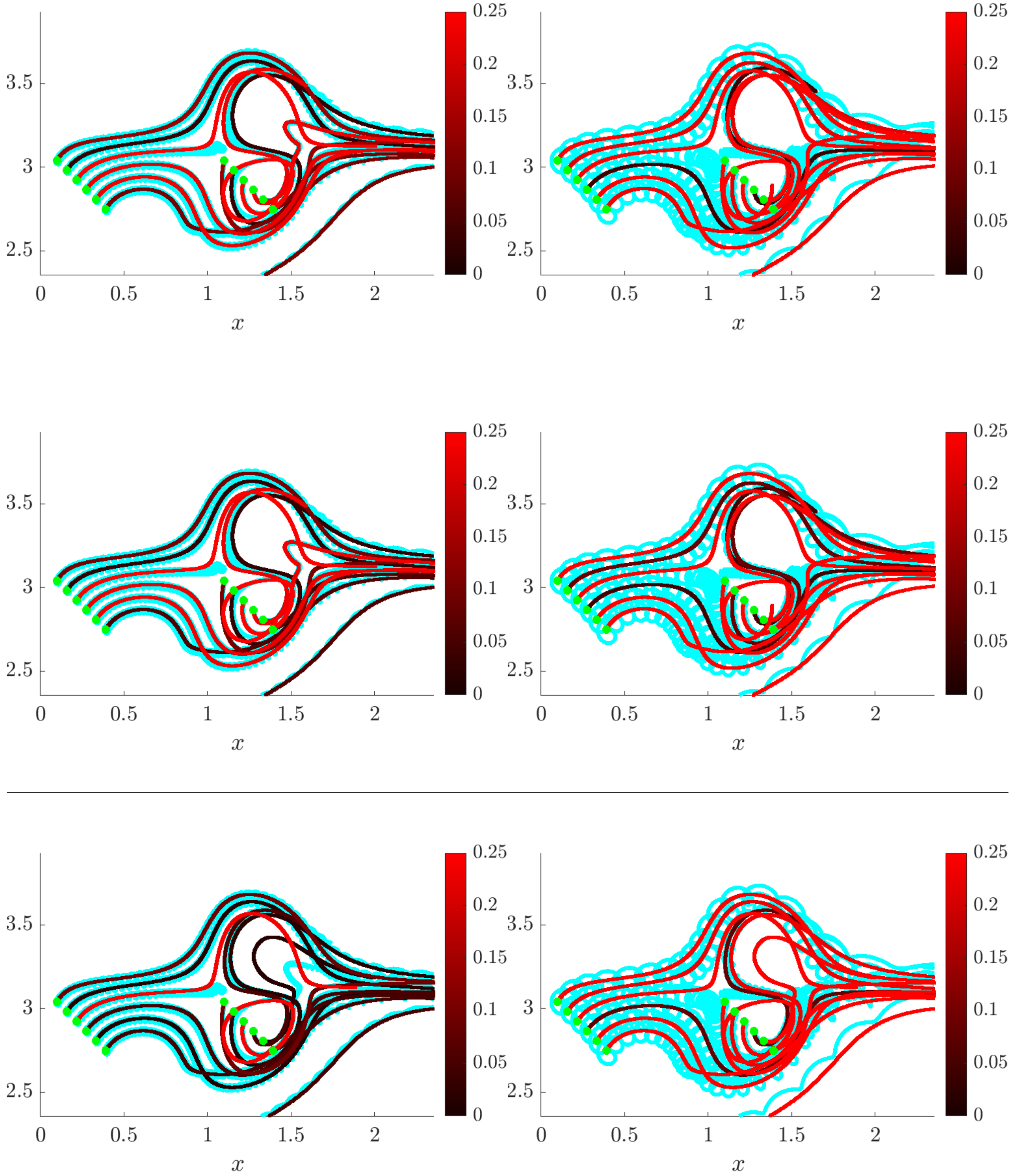

Figure S8. **Forward simulation accuracy for Example 4**. Same layout as Figure S5.

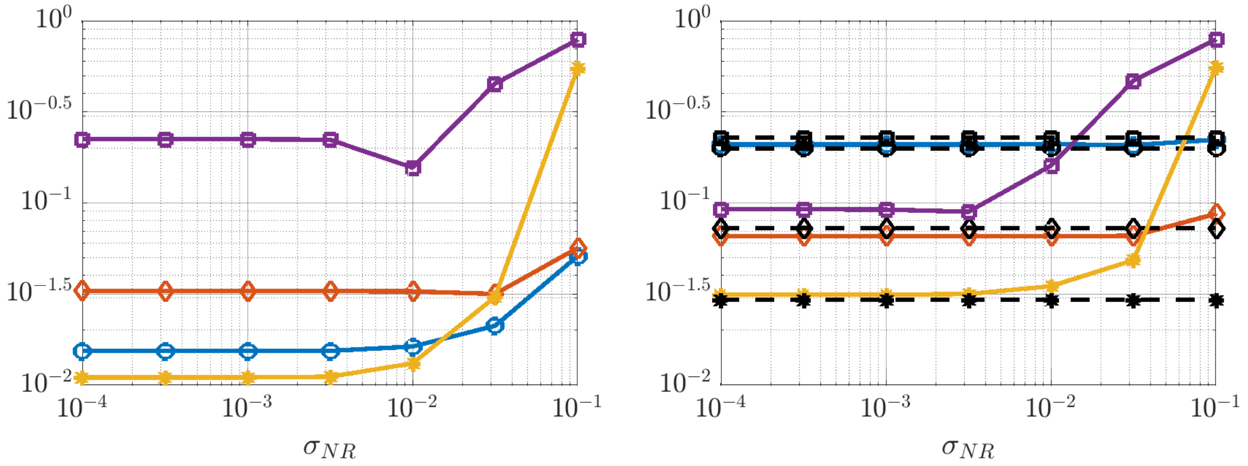

Figure S9. Forward simulation accuracy for noisy data. Left and right depict trends in $\Delta\mathbf{Z}^{\mu}$ and $\Delta\mathbf{Z}$, respectively. Results for Examples 1-4 are shown in blue, red, yellow and purple (as in Figure 10 in the main text). On the right values $\Delta\mathbf{Z}^{\star}$ are also shown in black for each example.

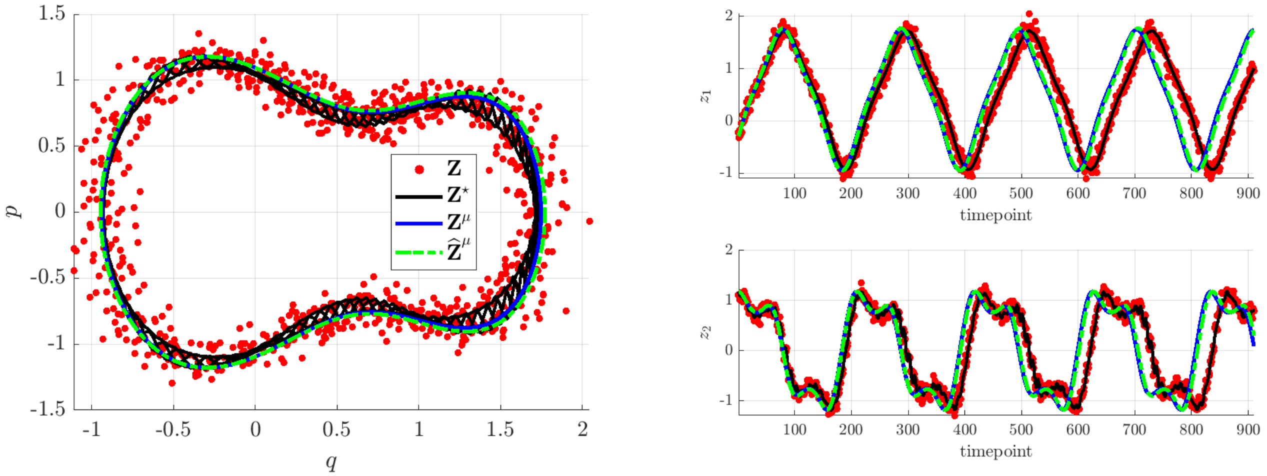

Figure S10. Visualization of recovery from Example 1 data with 10% noise. Images depict the noisy data used in training (red), corresponding clean data $\mathbf{Z}^{\star}$ (black), simulation from the true reduced system $\mathcal{H}_0^{\mu}$ (blue), and simulations from the learned model (green).

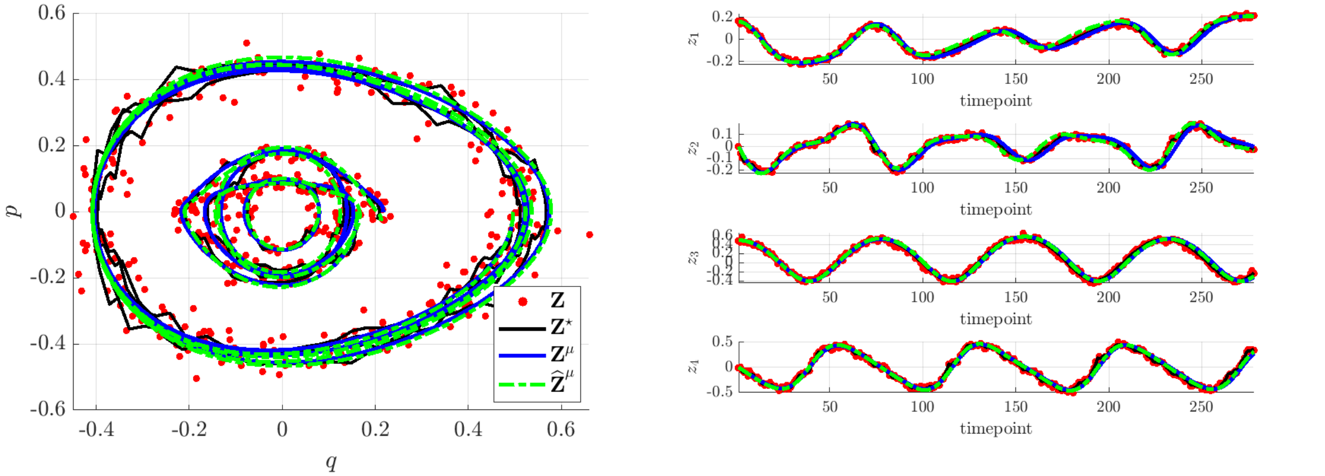

Figure S11. Visualization of recovery from Example 2 data with 10% noise. Images depict the noisy data used in training (red), corresponding clean data $\mathbf{Z}^\star$ (black), simulation from the true reduced system $\mathcal{H}_0^\mu$ (blue), and simulations from the learned model (green).

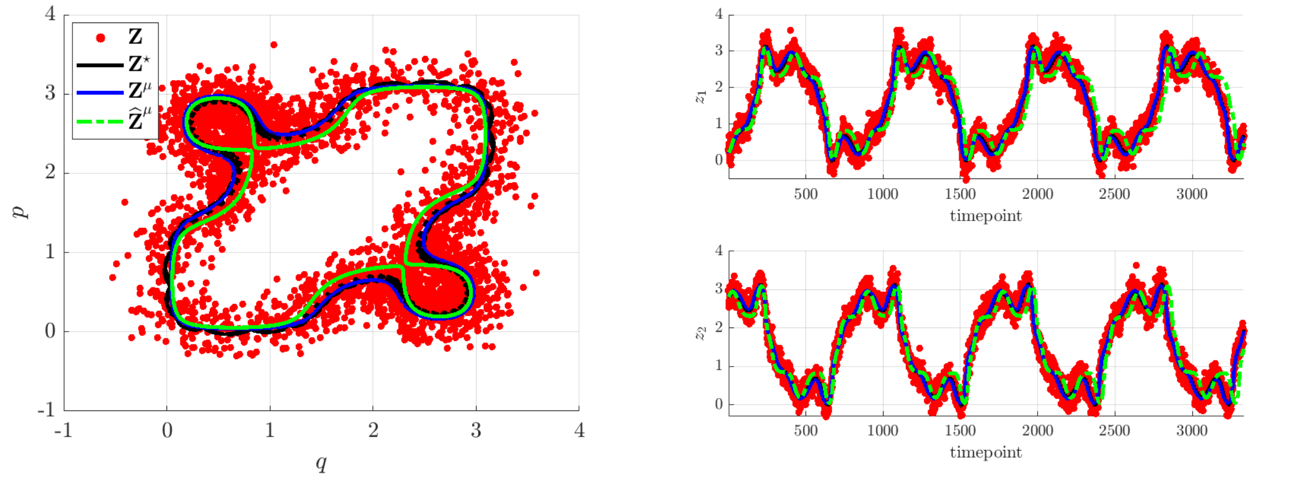

Figure S12. Visualization of recovery from Example 3 data with 10% noise. Images depict the noisy data used in training (red), corresponding clean data $\mathbf{Z}^\star$ (black), simulation from the true reduced system $\mathcal{H}_0^\mu$ (blue), and simulations from the learned model (green).

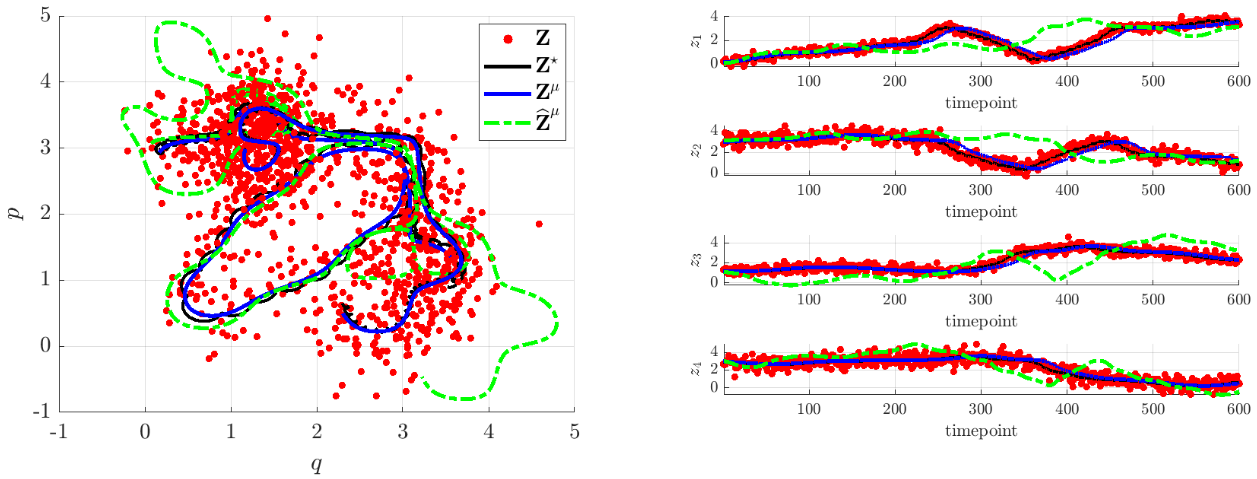

Figure S13. Visualization of recovery from Example 4 data with 10% noise. Images depict the noisy data used in training (red), corresponding clean data $\mathbf{Z}^\star$ (black), simulation from the true reduced system $\mathcal{H}_0^\mu$ (blue), and simulations from the learned model (green).
